# Characterization of the CsCENH3 protein and centromeric DNA profiles reveal the structures of centromeres in cucumber

**DOI:** 10.1093/hr/uhae127

**Published:** 2024-05-07

**Authors:** Yi Wang, Fang Zhou, Yangang Li, Xiaqing Yu, Yuhui Wang, Qinzheng Zhao, Xianbo Feng, Jinfeng Chen, Qunfeng Lou

**Affiliations:** State Key Laboratory of Crop Genetics and Germplasm Enhancement, College of Horticulture, Nanjing Agricultural University, Weigang Street No.1, Xuanwu District, Nanjing 210095, China; State Key Laboratory of Crop Genetics and Germplasm Enhancement, College of Horticulture, Nanjing Agricultural University, Weigang Street No.1, Xuanwu District, Nanjing 210095, China; State Key Laboratory of Crop Genetics and Germplasm Enhancement, College of Horticulture, Nanjing Agricultural University, Weigang Street No.1, Xuanwu District, Nanjing 210095, China; State Key Laboratory of Crop Genetics and Germplasm Enhancement, College of Horticulture, Nanjing Agricultural University, Weigang Street No.1, Xuanwu District, Nanjing 210095, China; State Key Laboratory of Crop Genetics and Germplasm Enhancement, College of Horticulture, Nanjing Agricultural University, Weigang Street No.1, Xuanwu District, Nanjing 210095, China; State Key Laboratory of Crop Genetics and Germplasm Enhancement, College of Horticulture, Nanjing Agricultural University, Weigang Street No.1, Xuanwu District, Nanjing 210095, China; State Key Laboratory of Crop Genetics and Germplasm Enhancement, College of Horticulture, Nanjing Agricultural University, Weigang Street No.1, Xuanwu District, Nanjing 210095, China; State Key Laboratory of Crop Genetics and Germplasm Enhancement, College of Horticulture, Nanjing Agricultural University, Weigang Street No.1, Xuanwu District, Nanjing 210095, China; State Key Laboratory of Crop Genetics and Germplasm Enhancement, College of Horticulture, Nanjing Agricultural University, Weigang Street No.1, Xuanwu District, Nanjing 210095, China

## Abstract

Centromeres in eukaryotes mediate the accurate segregation of chromosomes during cell division. They serve as essential functional units of chromosomes and play a core role in the process of genome evolution. Centromeres are composed of satellite repeats and highly repetitive centromeric retrotransposons (CRs), which vary greatly even among closely related species. Cucumber (*Cucumis sativus*) is a globally cultivated and economically important vegetable and the only species in the *Cucumis* genus with seven pairs of chromosomes. Therefore, studying the centromeres of the *Cucumis* subgenus may yield valuable insights into its genome structure and evolution. Using chromatin immunoprecipitation (ChIP) techniques, we isolated centromeric DNA from cucumber reference line 9930. Our investigation into cucumber centromeres uncovered the centromeric satellite sequence, designated as CentCs, and the prevalence of Ty1/*Copia* long terminal repeat retrotransposons. In addition, active genes were identified in the CsCENH3 nucleosome regions with low transcription levels. To the best of our knowledge, this is the first time that characterization of centromeres has been achieved in cucumber. Meanwhile, our results on the distribution of CentCs and CsCRs in the subgenus *Cucumis* indicate that the content of centromeric repeats in the wild variants was significantly reduced compared with the cultivated cucumber. The results provide evidence for centromeric DNA amplification that occurred during the domestication process from wild to cultivated cucumber. Furthermore, these findings may offer new information for enhancing our understanding of phylogenetic relationships in the *Cucumis* genus.

## Introduction

The centromere is a specific region of eukaryotic chromosomes that is crucial for the segregation and transmission of chromosomes during eukaryotic mitosis and meiosis. With the deepening of research, the function and structure of the centromere are gradually being understood [1, 2]. The centromere provides assembly and attachment sites for the kinetochores, which couple chromosomes to spindle microtubules, enabling the poleward movement of chromosomes [3, 4]. The replacement of canonical histone H3 in chromatin by centromere-specific histone H3 (CENH3) is a unique characteristic of centromeres [5]. CENH3 has a variable N-terminus, but its C-terminus is highly conserved [6, 7]. During cell division, only nucleosomes containing the CENH3 protein can become dynein aggregation sites to form the kinetochore [8, 9]. CENH3 can decipher centromeric DNA sequences because it binds exclusively to centromeric DNA [10].

In plants, the DNA sequences in the centromeric regions mainly consist of satellite repeats, centromeric retrotransposons (CRs), and other low-copy sequences. The length and distribution of satellite repeats are species-specific [7]. For instance, CentO satellite repeats consist mainly of 155- and 165-bp monomers, which have been identified throughout a super pan-genome in rice [11]. Additionally, the *Arabidopsis* pan-centromere was found to contain two satellite repeats, AthCEN178 (~178 bp, formerly known as CEN180) and AthCEN159 (159 bp, formerly known as CEN160), which are mainly monomers in *Arabidopsis* [12]. Similar findings have been reported in grapevines, which contain monomers of different lengths, including 107, 79, and 135 bp [13]. Moreover, the length of the centromeric satellite DNA varies even among different chromosomes of the same species, making the study of centromeric DNA more challenging [14, 15]. Transposable elements (TEs) are the predominant components of many genomes, accounting for 10–85% [16]. Neocentromeric satellites derived from TEs and other sequence repeats have been identified in a variety of species. For example, the centromeric repeat St3–294 identified in potatoes consists of an exceptionally long monomer, spanning 5.4 kb, and exhibits a high degree of similarity to the typical long terminal repeat (LTR) retrotransposable element [17]. In contrast, centromere analysis of the tetraploid cotton ancestral species *Gossypium raimondii* showed that CRs do not contain long arrays of centromeric satellites [18]. Moreover, Sharma *et al.* have characterized the tandem repeats CRM1TR and CRM4TR, which are derived entirely from uniparental centromeric retrotransposons in maize. These two repeats share a high degree of sequence homology (>95%) with extant retrotransposons [19]. Therefore, retrotransposons may be major contributors to the origin of novel centromeric satellites, and CRs have been identified across different species [20].

The latest preprint shows that RNAi is necessary for proper chromosome segregation at centromeres, creating a conflict between gene function and the role of the centromere [21]. However, studies on centromeric DNA of multiple species have revealed the presence of active genes in the centromeric regions. In *Arabidopsis*, transcription of more than 47 genes has been detected around the core centromeric region that contains CEN180 repeats [22]. In addition, there are 11 genes with transcriptional activity at the centromere of the maize chromosome [23], while the centromeres have at least 4 active genes in rice [11, 24]. Although telomere-to-telomere gene assembly has been completed in humans [25], maize [26], soybean [27], and other species [28, 29] in recent years, the complete assembly of centromeric regions, which are rich in satellite repeats, remains a challenge. Therefore, the identification and characterization of centromeres are of particular importance in most sequenced eukaryotic genomes.

The subgenus *Cucumis* comprises two species, namely *Cucumis sativus* (2*n* = 2*x* = 14) and *Cucumis hystrix* (2*n* = 2*x* = 24). *Cucumis sativus* exhibits three distinct variants, comprising the cultivar *C. sativus* var. *sativus* and two wild variants, *C. sativus* var. *xishuangbannanesis* and *C. sativus* var. *hardwickii*. In this study we characterized CsCENH3, a protein that can be utilized as a marker for the identification of centromeric sequences in cucumber. We performed immunostaining localization of CsCENH3 and fluorescence *in situ* hybridization (FISH) of ChIPed (ChIP, chromatin immunoprecipitation) DNA to examine the chromosomal localization of centromere-associated repeat sequences. ChIP sequencing (ChIP-seq) was employed to sequence CsCENH3-binding domains, enabling the identification of cucumber centromeres and the exploration of genes associated with centromeres. These studies elucidate differences in the distribution of centromere sequences within the subgenus *Cucumis*, offering valuable insights into the evolutionary mechanisms underlying centromere evolution in the subgenus *Cucumis*. Conducting a systematic investigation of centromeres will make a valuable contribution to enhancing the comprehensiveness of the genetic and physical mapping of the cucumber genome.

## Results

### Identification and characteristics of *CsCENH3* gene

**Figure 1:**
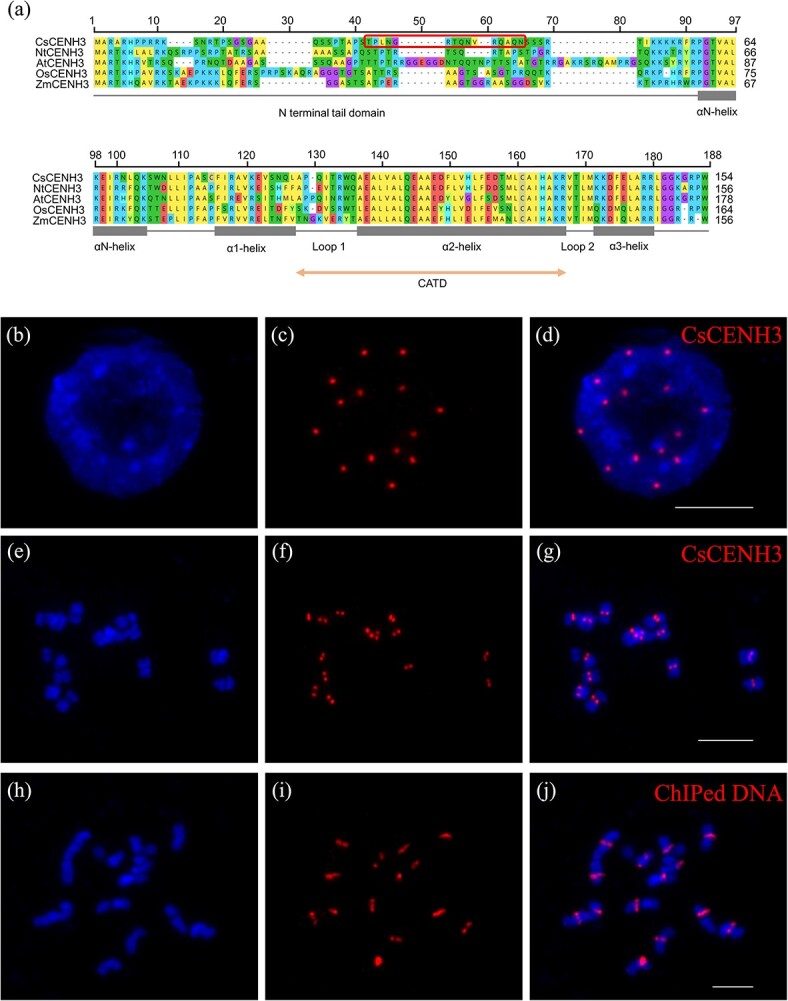
Alignment of CENH3 homologs and localization of CsCENH3 protein and ChIPed DNA on cucumber chromosomes. **a** Multiple alignment of CsCENH3 (*C. sativus*), NtCENH3 (*Nicotiana tabacum*), AtCENH3 (*A. thaliana*), OsCENH3 (*Oryza sativa*) and ZmCENH3 (*Zea mays*). The protein structure is shown below the sequence. The peptide sequence used to generate anti-CsCENH3 is highlighted in a red box. **b**–**g** Immunostaining localization of CsCENH3. Interphase nuclei and metaphase chromosomes (blue) were stained with 4′,6-diamidino-2-phenylindole (DAPI). Immunofluorescence signals (**c** and **d**, **f** and **g**) are visible within the nucleus (**d**) and in the centromeric regions of the chromosomes (**g**). **h**–**j** FISH signal of DNA precipitated by ChIP using anti-CsCENH3 antibody. Somatic metaphase chromosomes (**h** and **j**) that were hybridized to CsCENH3 ChIPed DNA probes. FISH signals (**i** and **j**) are visible in the centromeres (**j**). Scale bars: 10 μm.

To identify the *CENH3* gene in cucumber, we used *AtCENH3* (also referred to as HTR12, *AT1G01370.1*) in *Arabidopsis thaliana* as a query for a BLAST search. A putative CsCENH3 (GenBank*:* XP_011659153.1) was identified that is 61% identical to AtCENH3; it also showed 48, 47, and 65% similarity to the CENH3 proteins of rice (GenBank: NP_001407555.1), maize (GenBank: NP_001105520.1), and tobacco (GenBank: XP_016502108.1) (Fig. 1a). Further genome-wide searching in the cucumber reference genome 9930 v3.0 supported *CsCENH3* as a single-copy gene located on chromosome 7 (14 654 006–14 663 986 bp). Multiple alignments revealed that the C-termini of CENH3 are highly conserved, including the CATD (comprising loop1 and α2-helix), which are crucial for centromere targeting, while their N-terminal tail domains exhibit significant diversity. It is evident that the homology of CENH3 between monocotyledons and dicotyledons exhibits a consistent pattern.

### Preparation and specificity of anti-CsCENH3 antibody

The antigenic epitope of CsCENH3 protein was analyzed in order to investigate the correlation between CsCENH3 and centromeres of cucumber. The polyclonal antibody from rabbit serum was prepared using the 31st to 45th amino acids of the N-terminal region of the CsCENH3 protein (Fig. 1a). To determine the specificity of the antibody, immunostaining was performed on the root tips of cucumber. The immunostaining results demonstrated that the CsCENH3 antibody effectively produced distinct and intense fluorescence signals at the centromeric regions of cucumber somatic metaphase chromosomes (Fig. 1b–g). Immunostaining was also conducted on two wild cucumber variants, *C. s.* var. *xishuangbannanesis* and *C. s.* var. *hardwickii*. We observed distinct immunostaining signals localized at the centromere of the two variants (Supplementary Data Fig. S1).

### Isolation of centromere-specific repeat sequences in cucumber

We conducted ChIP on cucumber leaf tissue to extract DNA bound to CsCENH3. To assess the efficiency of ChIP, FISH technology was used to label the ChIPed DNA on the metaphase chromosomes of cucumber somatic cells. The results showed strong FISH signals at the centromeric regions of the chromosome (Fig. 1h–j), confirming the enrichment of centromeric DNA in the ChIPed DNA.

To identify the clusters of centromere-rich repeats, ChIP followed by ChIP-seq was performed using 150-bp paired-end reads. We obtained 12.93 and 15.61 million reads from ChIPed DNA and input DNA, respectively. A total of 500 000 reads were randomly selected from the input-seq data. Out of these, 344 040 reads were included in the cluster analysis. Among the participating reads, 302 206 reads (87.8%) formed 55 873 clusters, while the remaining 41 834 reads (12.2%) were identified as single-copy sequences (Supplementary Data Fig. S2). To calculate the ratio of ChIPed DNA reads to input DNA reads in each cluster, the ChIPed DNA reads and input DNA reads were aligned to the clusters. We selected seven clusters with ChIP/input ratios >1.7 for further analysis (Supplementary Data Fig. S3), five of which (CL3, CL10, Cs13, Cs17, and Cs66) showed noticeably higher ChIP/input ratios (>2), while CL231 and CL303 exhibited relatively high ChIP/input ratios (>1.7). From each cluster, the repeat exhibiting the highest read depth was subjected to PCR amplification for FISH analysis (Table 1).

**Table 1:** Statistical analysis of centromere-related repeat clusters in cucumber cultivar 9930

**Cluster**	**ChIP/genome ratio**	**Genome proportion (%)**	**Repeat**	**Length (bp)**	**Repeat type**
CL3	8.73	7.2	Cs3	177	satellite DNA
CL10	2.09	1.3	Cs10	2326	Ty3/*Gypsy*
CL13	2.05	0.8	Cs13	2113	Ty1/*Copia*
CL17	2.28	0.53	Cs17	1647	Ty1/*Copia*
CL66	2.47	0.04	Cs66	303	Ty1/*Copia*
CL231	1.78		Cs231	270	
CL303	1.73		Cs303	448	

Empty cells indicate that repeated sequences account for <0.01% of the genome, and the type is unknown.

**Figure 2:**
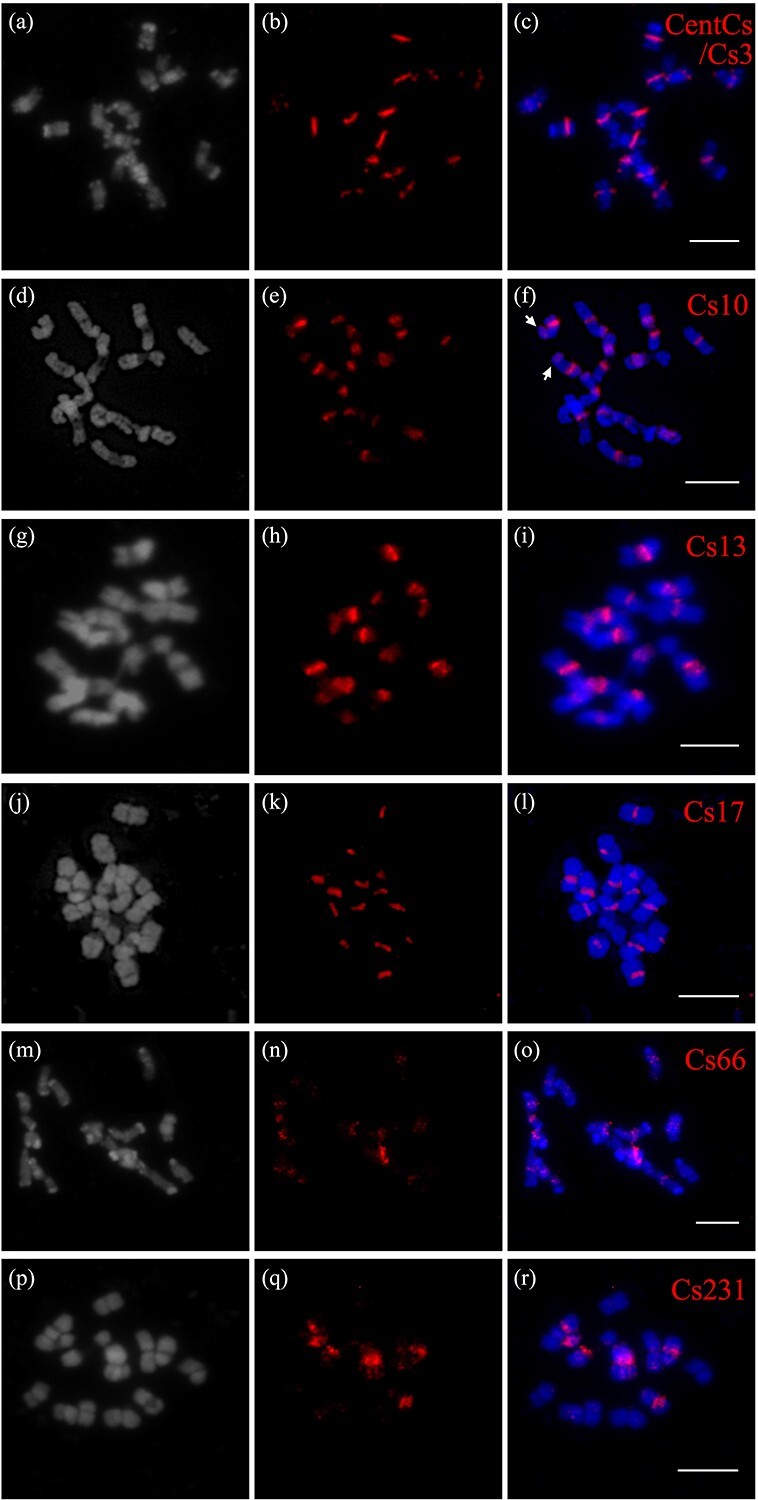
FISH mapping of centromeric repeats in cucumber cultivar 9930.** a**–**r** FISH signals (red) represent the CsCENH3-associated repeat cluster probes CentCs/Cs3 (**a**–**c**), Cs10 (**d**–**f**), Cs13 (**g**–**i**), Cs17 (**j**–**l**), Cs66 (**m**–**o**) and Cs231 (**p**–**r**) that stained with DAPI (gray and blue). Arrows (**f**) indicate the FISH signals of Cs10 on chromosome arms. Scale bar: 10 μm.

FISH analysis revealed that out of the seven repeats examined, four repeats (Cs3, Cs13, Cs17, Cs66) exhibited distinct signals at the centromeric regions of cucumber chromosomes (Fig. 2a–o); however, the signal intensity of Cs66 was comparatively weaker than the others (Fig. 2m–o). It is worth noting that Cs231 exhibited bright signals in the centromeric regions of three pairs of chromosomes (*cent1*, *cent4*, *cent5*) (Fig. 2p–r), while Cs303 did not display any FISH signal, attributed to its low genome proportion. Cs10 signals were observed to be present not only in the centromeric regions of all chromosomes but also in the arms of a few chromosomes (Fig. 2d–f, indicated with arrows). These non-centromere signals may be caused by the incomplete evolution of retrotransposons into CRs. Similar results have been reported in other species, such as rice [30] and cotton [18]. Based on these, the five repeat clusters (Cs3, Cs10, Cs13, Cs17, and Cs66) that produced relatively distinct centromeric FISH signals were used for subsequent analysis.

### Cucumber centromeres comprise satellite and retrotransposon-like repetitive sequences

**Figure 3:**
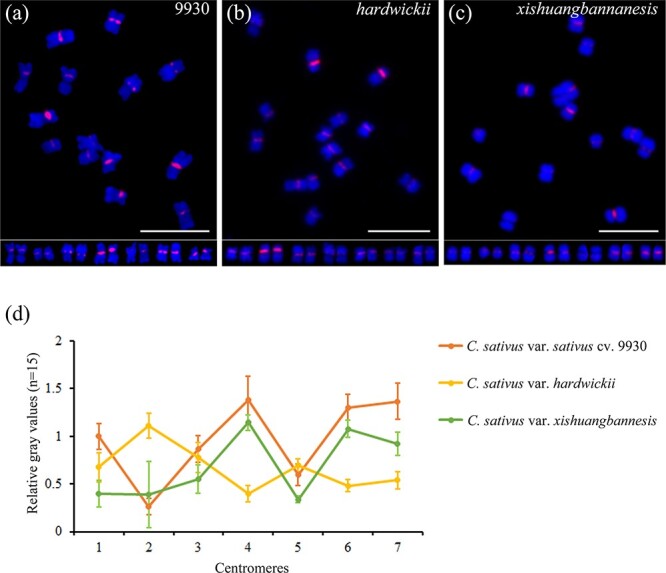
Comparative analysis of centromeric satellite CentCs by FISH and gray values in cucumber cultivar 9930 and two wild variants, *C. s.* var. *hardwickii* and *C. s.* var. *xishuangbannanesis*. **a*–*c** FISH mapping of the centromeric satellite CentCs in 9930 (**a**), *C. s.* var. *hardwickii* (**b**) and *C. s.* var. *xishuangbannanesis* (**c**); the arrangement of chromosomes is below each figure. Scale bars: 10 μm. **d** Difference in gray values of the CentCs FISH signal for each centromere (*n* = 15) in the three cucumber species.

To understand the origin of centromeric repeats, sequence analysis was conducted on five specific repeats. The repetitive sequence Cs3 exhibited 94.75% identity with the previously characterized satellite sequence type III (Supplementary Data Fig. S4a). Based on the distribution pattern and genomic proportion of Cs3, we determined that Cs3 corresponds to the centromeric satellite sequence in cucumber (CentCs). The monomer size of CentCs is 177 bp (Supplementary Data Fig. S4a). We also observed differences in the FISH signals of CentCs during somatic metaphase in seven pairs of chromosomes. For instance, *cent4*, *cent6*, and *cent7* exhibited elevated signal intensity, whereas *cent2* exhibited the lowest signal intensity (Figs 2a–c and  3a). In each chromosome, the intensity of the FISH signal corresponded to the copy number of CentCs.

The other four repeats, specifically Cs10, Cs13, Cs17, and Cs66, displayed retrotransposon-like features (Table 1, Supplementary Data Fig. S4b). According to the cluster analysis annotation, Cs10 showed similarity to the Ty3/*Gypsy*-like retrotransposon, while the other three repeats, Cs13, Cs17, and Cs66, exhibited a significant degree of sequence similarity (>90%; coverages were 42, 94, and 97%, respectively) to the Ty1/*Copia*-like retrotransposon [31] (GenBank: GQ326556.1) conserved among *Cucumis* species (Supplementary Data Fig. S4b). Apart from the FISH signal in centromeric regions, dispersed signals of Cs10 were also observed in the non-centromeric regions, indicating that the Ty3/*Gypsy* class retrotransposon, to which Cs10 belongs, may have originated from chromosome arms. FISH signals from three CRs belonging to the Ty1/*Copia* class showed centromere-specific distribution patterns. It is possible that they originated from centromeric retrotransposons in *Cucumis*. Our results show that most of the CR elements in cucumber belong to Ty1/*Copia* retrotransposons, and the same is true in sacred lotus [32] and banana [33].

### Cucumber centromeres contain a significant amount of repetitive sequence

Our clustering results showed that the CentCs accounted for 7.2% (16 272 kb) of the cucumber genome (Supplementary Data Fig. S5, Table 1). This percentage is similar to the CEN180 monomers found in *Arabidopsis* (8.8%) [34], suggesting a significant presence of satellite DNA in the centromeric regions. The results of the BLAST alignment showed that most of the 177-bp monomers were located in the proximal centromeric regions of cucumber. A total of 4292 copies of CentCs were identified, with a total length of 760 kb, significantly deviating from the predicted length (16 272 kb). It is worth noting that a total of 97 copies of CentCs were identified on chromosome 3, which was inconsistent with the expected value based on the FISH signal. This phenomenon was frequently observed in centromeric regions that exhibited high levels of repetition [17].

The comprehensive genome-wide examination of centromeric retrotransposons in cucumber (CsCRs) unveiled an intriguing finding: while primarily situated in the putative centromeric regions, they also appear along the chromosome arms. This distribution pattern stands in stark contrast to the centralized positioning typically observed for CentCs (Supplementary Data Fig. S5). The current version (v3.0) of the cucumber genome contains multiple scaffolds that have not been assembled onto the chromosomes, suggesting that the copy numbers of CentCs and CsCRs were underestimated in the genome assembly for the cultivated cucumber 9930.

### Evolutionary divergence of centromere sequences in subgenus *Cucumis*

To investigate the variations in CentCs among different wild cucumber variants and cultivars, an analysis of gray values was conducted on the CentCs FISH signals of the inbred cucumber line 9930 and two wild cucumber variants (Fig. 3, Supplementary Data Table S2). The signal intensity from the centromeric satellite CentCs showed distinct differences among them. The CentCs signal of *cent4* in 9930 and *C. s.* var. *xishuangbannanesis* exhibited the highest intensity, whereas the *cent4* signal in *C. s.* var. *hardwickii* exhibited the lowest level of strength. The CentCs signal of *cent2* exhibited the highest intensity in *C. s.* var. *hardwickii*, but it was relatively weak in 9930 and *C. s.* var. *xishuangbannanesis* (Fig. 3a–d). Furthermore, notable variations were observed in the grayscale values of the remaining centromeres, indicating significant differences among them. For example, the gray value of *cent1* of 9930 was 2.5 times that of *C. s.* var. *xishuangbannanesis* (Fig. 3d). Hence, the fluctuating FISH signal patterns of CentCs suggest that species belonging to the subgenus *Cucumis* exhibit significant variations in the amplification or deletion of centromeric satellite copy numbers.

**Figure 4:**
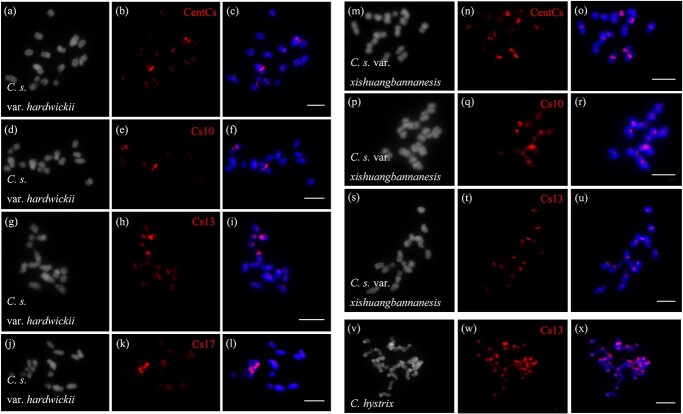
FISH determined the distribution of CsCRs in the subgenus *Cucumis* species. **a**–**x** CsCRs probes were hybridized to somatic metaphase chromosomes of *C. s.* var. *hardwickii* (**a**–**l**), *C. s.* var. *xishuangbannanesis* (**m**–**u**), and *C. hystrix* (**v**–**x**). Scale bars: 10 μm.

We conducted FISH experiments on the somatic metaphase chromosomes of two wild cucumber variants, utilizing four CsCRs probes (Cs10, Cs13, Cs17, and Cs66) (Fig. 4, Table 2). The results demonstrated that Cs10, Cs13, and Cs17 were all distributed in the centromeric region of *C. s.* var. *hardwickii*, but they were only present in specific chromosomes (Fig. 4d–l). Compared with the cultivated cucumber 9930, Cs10 and Cs13 exhibited similar distribution patterns in *C. s.* var. *xishuangbannanesis* (Fig. 4p–u). No signal of Cs66 was detected in two wild cucumber variants, indicating its relatively small proportion within the genome. In addition, we conducted hybridization of CsCRs with the somatic metaphase of *C. hystrix*, but no signal was observed at its centromeres. Only Cs13 exhibited bright signals at the telomeres of *C. hystrix* chromosomes (Fig. 4v–x), indicating the possibility of chromosomal rearrangements taking place in *C. hystrix* and *C. sativus* during their extensive evolutionary process. In brief, the repetitive sequences observed in this study consisted of not only satellite DNA but also centromeric retrotransposons. It was found that these repetitive sequences were less abundant in the wild variants compared with the cultivated cucumber. It is hypothesized that during the domestication process of wild species there is a tendency to an increased frequency of repetitive sequences.

**Table 2:** FISH signal distributions of centromeric repeat sequences in subgenus *Cucumis*

**Repeat**	**Taxon**			
	**9930**	***C. s.* var. *hardwickii***	***C. s. var. xishuangbannanesis***	***C. hystrix***
Cs3/CentCs	CRA	CRA	CRA	
Cs10	CRA and telomeric regions of two pairs of chromosomes	CRA	CRA and bright signals on centromeric regions of two pairs of chromosomes	
Cs13	CRA	Bright signals on centromeric regions of two pairs of chromosomes, dispersed signals on the others	CRA	Telomeric regions of all chromosomes
Cs17	CRA	Dispersed weak signals on all chromosomes and bright signals on centromeric regions of one pair of chromosomes		
Cs66	CRA			
Cs231	Centromeric regions of three pairs of chromosomes			
Cs330				

CRA, centromeric regions of all chromosomes.

Empty cell: no detectable FISH signals.

### Centromere size and assembly in cucumber

**Figure 5:**
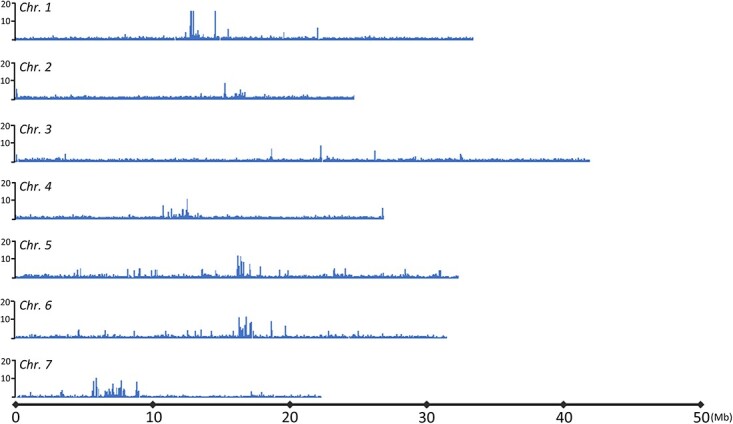
Identification of CsCENH3-binding regions on each chromosome of cucumber. The adjusted ChIP-seq reads were mapped to the reference genome of 9930. The *x*-axis represents the position on the chromosome and the *y*-axis represents read density. Read density was expressed as the number of mapped unique reads for the corresponding 10-kb window.

To unveil the association between centromeric regions and CsCENH3, we adopted the ChIPed DNA sequence mapping method. A total of 12.93 and 15.61 million 150-bp paired-end reads were obtained for the ChIPed DNA and input DNA, respectively. Among them, 2.26 million reads were successfully aligned to the genome assembly 9930 v3.0. The chromosomes were divided into 10-kb windows, and relative enrichment was calculated as the ratio of reads from ChIPed DNA to reads from input DNA for each window. This analysis resulted in the identification of peaks corresponding to nucleosome regions bound by the CsCENH3 protein on six out of the seven cucumber chromosomes (Fig. 5, Table 3). The six centromeres that have been annotated in the cucumber genome exhibited varying sizes, with *cent5* measuring 0.3 Mb and *cent4* measuring 0.75 Mb. Collectively, these centromeres accounted for 1.92% of the cucumber genome. The missing CsCENH3-binding domain on chromosome 3 could potentially be attributed to misassembly (Fig. 3a), a phenomenon frequently observed in highly repetitive regions.

**Table 3:** Summary of CENH3-binding regions in cucumber cultivar 9930

**Chromosome**	**CsCENH3-binding region**								
		**Start**	**End**	**Length**	**Total length**	**Chromosome length**	**Proportion** ^**a**^	**No. of**	**No. of genes in**
		**(Mb)**	**(Mb)**	**(kb)**	**(kb)**	**(Mb)**	**(%)**	**genes**	**CsCENH3 subdomains**
1	Cent1_1	12.5	12.8	289.5	438.4	32.9	1.33	13	1
	Cent1_2	14.3	14.4	148.9				9	2
2	Cent2	15.8	16.4	557.9	557.9	24.8	2.25	31	4
4	Cent4	11.5	12.2	750.8	750.8	26.8	2.80	1	0
5	Cent5	15.9	16.2	299.5	299.5	31.9	0.94	3	0
6	Cent6	15.9	16.5	582.2	582.2	31.1	1.87	31	2
7	Cent7_1	6.0	6.5	449.6	636.2	22.5	2.83	32	5
	Cent7_2	7.7	7.9	186.6				13	1
Total					3265.0	170.1	1.92	133	15

a Centromere length/chromosome length × 100.

### CENH3 and H3 subdomains interspersed in cucumber centromeres

**Figure 6:**
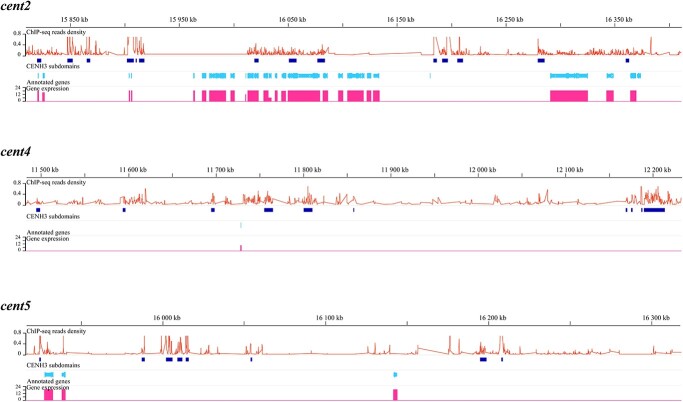
Localization of CsCENH3-binding domains and gene expression in centromeres 2, 4, and 5. The top track represents the physical location of the corresponding chromosome. ChIP-seq read densities were calculated in 1-kb windows and are represented by line plots. CsCENH3 subdomains are represented by blue bars. Annotated genes are shown in coordination with the cucumber genome (light blue). Pink vertical bars represent the number of tissues in which the gene is expressed.

To distinguish between the CsCENH3 subdomains and the CsH3 subdomains, we utilized the SICER algorithm [35] to differentiate the CsCENH3 and CsH3 subdomains identified in the six centromeres of the cucumber genome. The distribution of CsCENH3 and CsH3 subdomains in cucumber centromeres was observed to be scattered (Fig. 6, Supplementary Data Figs S6 and S7). Our results indicate that CsCENH3 subdomains varied in size, ranging from 1 to 26 kb. There are multiple large CsH3 subdomains (>50 kb) in the centromeric regions of certain cucumber chromosomes. It is evident that *cent1* and *cent7* were composed of two distinct CsCENH3-binding domains, separated by a significantly large CsH3 subdomain (1.44 and 1.24 Mb, respectively). In addition, it was observed that *cent6* and *cent7* possessed CsH3 subdomains that contain multiple genes, which have also been identified in other crops [36].

### Low gene density and transcriptional activity in the CsCENH3-binding regions

According to the cucumber 9930 genome annotation database, 133 non-TE genes were identified in the CsCENH3-binding regions. Fifteen of these genes (11%) were located in the CsCENH3 subdomain, while the remaining 118 genes (89%) were located in the CsH3 subdomain (Fig. 6, Supplementary Data Figs S6 and S7, Supplementary Data Table S3). The gene density in the cucumber centromere is ~25 kb per gene, which is significantly lower than the average gene density of the entire genome (9.3 kb per gene). At the subdomain level, the gene density within the CsCENH3 subdomains is notably lower than that in the CsH3 subdomains. This distinction was clearly evident in the CsCENH3-binding domains of *cent2*, *cent6*, and *cent7*.

**Figure 7:**
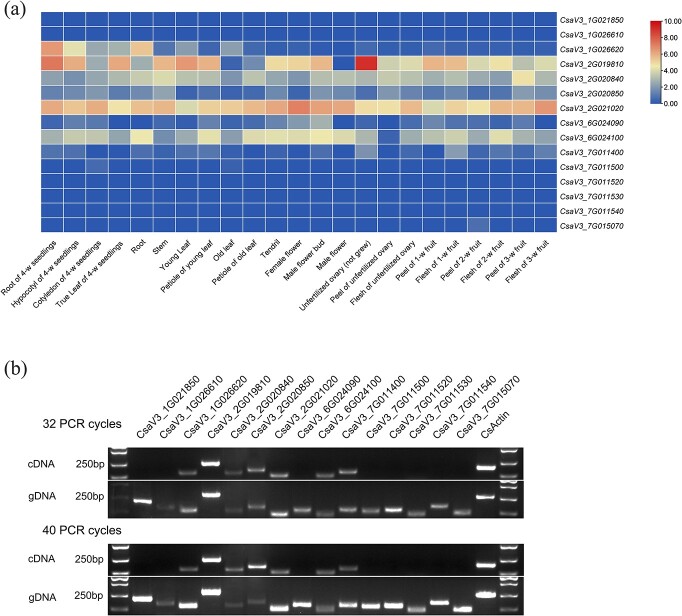
Transcriptional analysis of CsCENH3 subdomain-related genes.

We then utilized public RNA-seq data (PRJNA312872) [37] conducted for 23 tissues, including leaves, male flowers, and female flowers, to investigate the transcriptional activity of centromeric genes (Fig. 6, Supplementary Data Figs S6 and S7, Supplementary Data Tables S3 and S4). Of the 15 genes located in the CsCENH3 subdomains, 10 were expressed in at least one tissue. Gene *CsaV3_7G011500* was weakly expressed in seedling cotyledons and old leaves [fragments per kilobase of exon model per million mapped (FPKM) <1], while gene *CsaV3_7G015070* exhibited a low expression level (FPKM = 0.67) only in 2-week-old fruit peels. Despite being expressed in multiple tissues, *CsaV3_2G020850*, *CsaV3_6G024090*, and *CsaV3_7G011400* also showed low expression levels (FPKM <5). The remaining five genes (*CsaV3_1G026620*, *CsaV3_2G019810*, *CsaV3_2G020840*, *CsaV3_2G021020*, *CsaV3_6G024100*) had relatively higher expression (FPKM >10) in multiple tissues (Fig. 7a, Supplementary Data Table S5). By contrast, the majority of genes (103 out of 118) located within CsH3 subdomains were expressed in at least one tissue (Supplementary Data Table S3).

We used reverse transcription PCR (RT–PCR) to validate the expression of these 15 genes in cucumber leaf tissues (Fig. 7b, Supplementary Data Table S5). The results showed that only seven genes were transcribed in leaves. Notably, *CsaV3_6G024090*, with lower transcriptional activity (FPKM = 0.17), was not detected. On the contrary, *CsaV3_7G011400* was not expressed in the RNA-seq data; however, it was detected in the cDNA extracted from the leaves. According to gene annotation (Supplementary Data Table S4), we speculate that *CsaV3_7G015070* is a pseudogene, as it was not detected by RT–PCR, and its mRNA length is only 156 bp. We performed quantitative ChIP–PCR (ChIP–qPCR), and the results confirmed the enrichment of these 15 genes in the ChIPed DNA (Supplementary Data Fig. S8). Our results confirmed the existence of these gene sequences in the CsCENH3 subdomain. In summary, gene density and activity decreased in the centromeric region, and the CENH3 subdomain contains fewer active genes than the H3 subdomain in cucumber.

## Discussion

Centromeres in most higher eukaryotes consist of satellite DNA and highly repetitive retrotransposon-like sequences [38]. The satellite DNA arrays not only have the capacity to fully occupy all CENH3 domains in centromeres but also to extend into the juxtacentromeric regions [39]. Centromeric satellite repeats exhibit rapid evolutionary changes, leading to the presence of distinct repeats that may occur in the centromeres of closely related species [40]. With the advances in sequencing technology, achieving telomere-to-telomere genome assembly for species is becoming increasingly feasible. The processing of centromeric region sequences is challenging due to their significant diversity. As a result, centromeres are often regarded as the final frontier in the genomes of multicellular eukaryotes [41]. Being the first fully sequenced vegetable crop, the distribution pattern and sequence composition of centromeres in cucumber have rarely been reported [42]. Our centromeric DNA profile provides us with a valuable strategy to study the structure, function, and evolution of cucumber centromeres.

In this study, we employed a clustering method based on sequence similarity to analyze the composition of repetitive DNA [43]. We identified CentCs and the CsCRs in cucumber. Cs3 was characterized as the cucumber centromeric satellite sequence CentCs, with a length of 177 bp. Like other eukaryotic centromeres, the CentCs, which are the dominant satellites, underwent rapid evolution and were found throughout the entire genome of cucumber. Previous research has indicated that retrotransposons are the main source of origin of neocentromeric satellite repeats [16]. We have identified several CsCRs, namely Cs10, Cs13, Cs17, Cs66, and Cs231, within the cucumber genome (Fig. 2d–r). The distribution of CsCRs exhibited diverse patterns. (i) Cs10 showed similarity to the Ty3/*Gypsy* class of retrotransposons (Table 1, Supplementary Data Fig. S4b). It displayed a bright signal not only in the centromeric regions but also in certain chromosome arms (Fig. 2d–f). This distribution pattern was also found in cotton [18] and sacred lotus [32]. (ii) Cs13, Cs17, and Cs66 were identified as centromere-specific repeats with the Ty1/*Copia* retrotransposon type (Fig. 2g–o, Supplementary Data Fig. S4b, Table 1). (iii) Cs231 was identified as a chromosome-specific repeat, exhibiting signals exclusively in the centromeric regions of three pairs of chromosomes (Fig. 2p–r). More interestingly, there are two types of CR in cucumber, which differ from those found in other crops. For example, the CRs in cotton [18] and bread wheat [44] all belong to the Ty3/*Gypsy* group, while the CRs of sacred lotus [32], a basal dicotyledonous sacred plant, are all of the Ty1/*Copia* type. The results indicated centromeric preference in the distribution of Ty1/*Copia* retrotransposons (CL13, CL17, CL66) in the cucumber genome. Research has demonstrated that satellite repeats can also be amplified from a small subset of retrotransposons [45]. Hence, it is plausible that retrotransposon-derived amplified DNA serves as a prevalent origin of centromeric DNA in different higher eukaryotes [20].

Genome variation is the foundation of genetic diversity within species, manifested in variations in DNA sequence and gene function [46, 47]. The intraspecific distribution of the centromere satellite CentBd in *Brachypodium distachyon* shows that the copy number of the centromere satellite has experienced different degrees of expansion or deletion, even within closely related inbred lines [36]. The subgenus *Cucumis* contains two diploid species, with basic chromosome numbers of 7 and 12. In this study, both CentCs and CsCRs were present in cucumber cultivar 9930 and two cucumber wild variants (Figs 3 and 4). We analyzed CentCs in the subgenus *Cucumis* for the first time, and the results showed that their CentCs contents on the same chromosome were significantly different. In general, CentCs content of two wild variants was lower than that of cultivated cucumber (Fig. 3). Notably, the distribution patterns of CsCRs were also quite different. The CsCRs signal distributions of *C. s.* var. *xishuangbannanesis* were found to be similar to those of cultivated cucumber; however, the copy number was smaller in *C. s.* var. *xishuangbannanesis*. Different from cultivated cucumber, CsCRs were found to be localized in the centromeric regions of the specific pairs of chromosomes in *C. s.* var. *hardwickii*. This observation suggests that the centromeric sequence of cucumber has undergone distinct evolutionary changes within the species. Thus, it supports the idea that the centromeric DNA of cucumber has experienced amplification during the domestication of cultivated cucumber. Nevertheless, *C. hystrix* did not contain CentCs and only one CsCR probe has signals on its telomeres (Fig. 4v–x). This provides evidence for the distinct origins of the diploid species *C. hystrix* and *C. sativus*. Sequence alignment and western blot analysis revealed high sequence similarity (98%) between the CENH3 sequences of *C. sativus* and *C. hystrix*, with both sequences exhibiting identical sizes (Supplementary Data Figs S9 and S10). Surprisingly, immunostaining signals were not detected in *C. hystrix* (Supplementary Data Fig. S1). We hypothesize that the difference in the three-dimensional structures of the CENH3 protein could impede the antibody’s ability to identify the target antigen, which needs further experiments for validation.

Neocentromeres often occur in gene-poor regions because centromeric chromatin is known to be incompatible with transcription [40, 48]. The chromatin environment required for transcription is determined by DNA methylations and histone modifications [49]. Due to the presence of CENH3 and a variety of histone modifications that inhibit transcriptional activity, the DNA at the centromeric region shows high methylation and contains a great number of TEs [50, 51]. Thus, there is a transcriptional incompatibility in the presence of CENH3, which inhibits the transcriptional activity of genes within the centromere. However, genes with transcriptional activity have been identified in the centromeric regions of various plant species, including sacred lotus [32], wheat [44], and other plants [18, 36]. In our study, a total of 15 genes were identified in the CsCENH3 subdomains and seven genes were found to be transcriptionally active, as confirmed by both RT–PCR and ChIP–qPCR (Fig. 7, Supplementary Data Fig. S8). Recently, researchers identified a centromeric gene, *AGIS_Os12g018490* (*OsMAB*), using eQTL and haplotype analysis, which plays a role in regulating tiller number in rice [11]. Several studies have also reported transcriptional activity in centromeric regions in plants and other species [52–54]. This indicates that the CsCENH3 binding is not completely incompatible with gene transcription. This result is not unreasonable because centromeres do not function throughout the entire cell cycle, which creates opportunities for gene transcription. Nevertheless, it is currently unclear whether alterations in these genes have an impact on the processes of chromosome segregation and plant growth. With the popularization of CRISPR/Cas9-based gene editing systems, these mysterious genes will surely be revealed in future studies.

Herein, we analyzed the composition of cucumber centromeres in detail by characterizing CsCENH3 and constructing centromeric DNA profiles. Our results identified and characterized cucumber centromeric elements – CentCs and CsCRs. We also investigated the evolutionary relationship of centromeres within the subgenus *Cucumis*. The results revealed significant differences in the content and distribution of CentCs and CsCRs within the subgenus, and CentCs and CsCRs of cultivated cucumber exhibited noticeable amplification compared with wild varieties. Taken together, this study may not only provide valuable insights into the evolution of cucumber centromeres, but also offer possibilities for improving the genetic map of cucumber and innovating germplasm resources.

## Materials and methods

### Plant materials

The inbred cucumber line 9930 (*C. sativus* var. *sativus* cv. 9930) was used as the material for ChIP experiments and cytological analysis. Two wild variants of cucumber, namely *C. s.* var. *xishuangbannanesis* and *C. s.* var. *hardwickii*, and a wild cucumber species, *C. hystrix*, were used in cytological experiments. All plants were grown at the Baima Teaching Base of Nanjing Agricultural University.

### Preparation of anti-CsCENH3 antibody

Anti-CsCENH3 polyclonal antibodies were generated using the synthetic peptide (TPLNGRTQNVRQAQN), which is located at the 31st to 45th amino acid residues of the predicted CsCENH3. The antibodies were custom synthesized and purified by Chemgen Co., Ltd (http://www.chemegen.net/).

### ChIP and ChIP-seq

ChIP assays were performed using CsCENH3 antibodies as previously described, with modifications [55]. Young leaves of cucumber (10 g) were ground into a fine powder in liquid nitrogen. Chromatin was extracted with nuclear lysis buffer and fragmented using MNase (micrococcal nuclease). Fragmented chromatin was subjected to immunoprecipitation (ChIP). ChIPed DNA and input DNA were submitted to Annoroad Gene Technology Co., Ltd for library construction and sequencing.

### ChIP-seq and RNA-seq mapping

Sequence reads from ChIP and input samples were first processed using FastUniq [56] and Trimmomatic [57] to remove PCR duplicates and low-quality reads. Using the Bowtie2 program [58], the data were aligned to the 9930 v3.0 genome assembly (http://cucurbitgenomics.org/). We allowed 2-bp mismatches and retained only uniquely aligned reads. Then, we divided the genome into 10-kb windows and counted the number of uniquely aligned reads within them. We adjusted the read density using the input data to reduce background noise. To identify the CENH3 domains of cucumber, ChIP-seq data were analyzed by SICER 1.1 [35], using the parameters described by Zhu *et al.* [32]. For genes that are not completely included in the CsCENH3 subdomain, if at least 50% of its sequence is located within the CsCENH3 subdomain, we define the gene to be located in the CsCENH3 subdomain.

We calculated the expression of genes located in the CsCENH3 binding domains using public transcriptome data containing 23 different tissues or organs of cucumber [37] (PRJNA312872) to find out the expression patterns of centromeric genes. RNA-seq analysis was conducted for the 23 sampled cucumber tissues following the published protocol [59]. First, we built a reference index using Bowtie2-build [58]. The cleaned raw reads were then aligned to the reference index using TopHat. Uniquely mapped reads with a mapping quality threshold >15 were filtered (*P* < 0.05). Transcripts were assembled using Cufflinks according to the cucumber annotation v3.0 (http://cucurbitgenomics.org/). Gene expression was estimated using the FPKM method. We used the default parameters for all the described software. Heat maps of genes within the CsCENH3 subdomains were visualized by TBtools-II [60].

### Identification of centromeric repeats

To identify centromere-associated repeats, we used a previously described similarity-based clustering method [17]. First, 500 000 reads were randomly selected from input data for graph-based cluster analysis, the data were uploaded to REPATEXPLORER, and default parameters were used to obtain duplicate clusters. Then, local BLAST tools were used to map ChIP-seq reads and input-seq reads to duplicate clusters with an e-value threshold of 1e−8. Based on the relative enrichment of each cluster (the ratio of ChIP-seq reads to input-seq reads per cluster), clusters with higher ratios were considered as potential centromeric repeats for FISH analysis.

### FISH and chromosomal immunofluorescence

The FISH procedure was adapted from a published protocol [61]. To compare signal intensities, 50 ng of each labeled probe was used in the FISH experiments, the same exposure time was set and then all images were captured on an Olympus BX51 microscope. The list of primers used in probe synthesis is provided in Supplementary Data Table S1. An immunostaining assay was carried out following the established protocol [62].

### RT–PCR and ChIP–qPCR

RT–PCR was used to detect the transcription levels of CsCENH3-associated genes. Total RNA was isolated from the leaves of cucumber. RT–PCR was conducted according to standard protocols. The numbers of amplification cycles of RT–PCR were 32 and 40 to distinguish those genes with low transcriptional activity. PCR was performed by the following procedure: 5 min at 95°C, then 32 or 40 cycles of 30 s at 94°C, 30 s at 60°C and 10 s at 72°C, and final extension at 72°C for 5 min. PCR products were analyzed using agarose gel electrophoresis.

We used ChIP–qPCR to verify the relative enrichment of 15 genes located in the CsCENH3 subdomain in the ChIP-seq data. The PCR reaction was performed with an iQ1 Real-time PCR system (Bio-Rad). *CsActin* (*CsaV3_2G018090*) was used as an internal reference. Each PCR reaction was performed in triplicate. The relative fold enrichment was calculated based on the 2^−ΔΔCt^ method. The list of primers is provided in Supplementary Data Table S5.

## Supplementary Material

Web_Material_uhae127
