# Supplementary material for: Characterization of the CsCENH3 protein and centromeric DNA profiles reveal the structures of centromeres in cucumber
Source: Hortic Res. 2024 May 7;11(7):uhae127. doi: 10.1093/hr/uhae127 (PMC11220175; doi:10.1093/hr/uhae127)
Supplement: Web_Material_uhae127 [file web_material_uhae127.zip › Supplementary figures.docx]

**Supplementary Information**


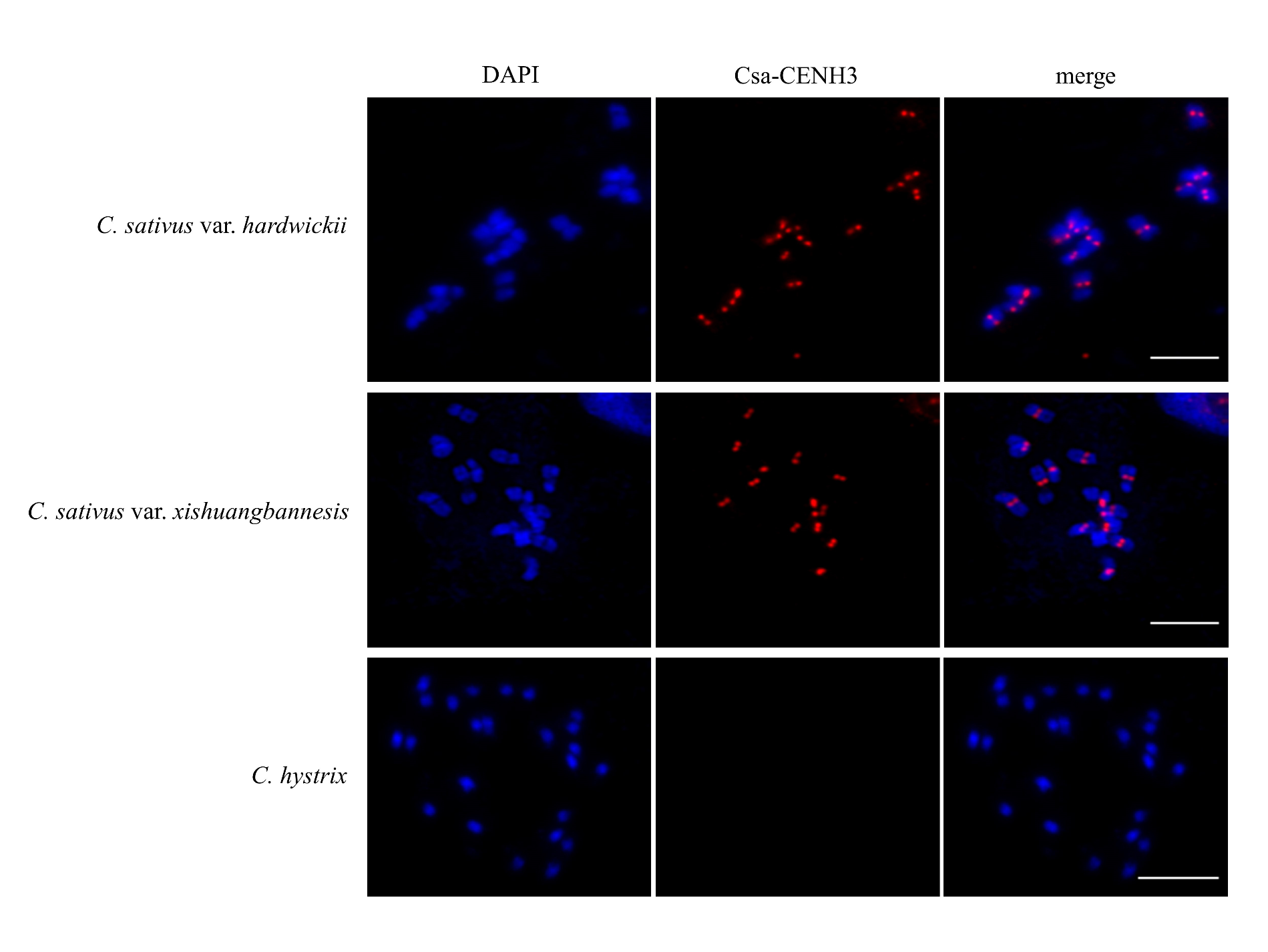


**Figure S1.** Immunostaining of anti-CsCENH3 antibody on somatic metaphase chromosomes of *C. sativus* var. *hardwickii*, *C. sativus* var. *xishuangbannesis* and *C. hystrix*. Scale bars = 10 µm


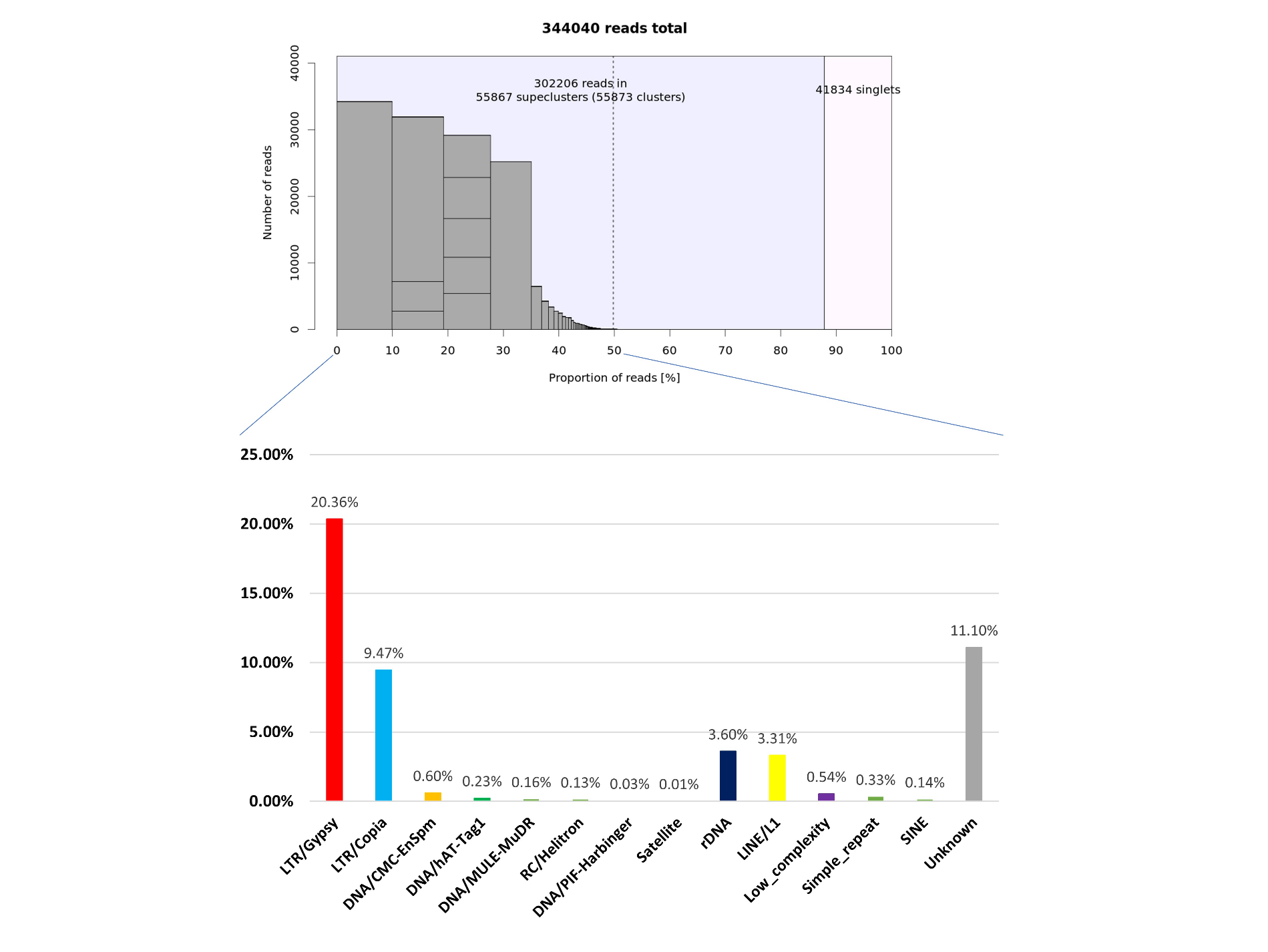


**Figure S2.** Identification of repetitive DNA in cucumber cultivar ‘9930’. The figure above summarized the content of each repeat family and single-copy reads. Annotation and genome percentage of duplicate clusters were shown in the figure below.


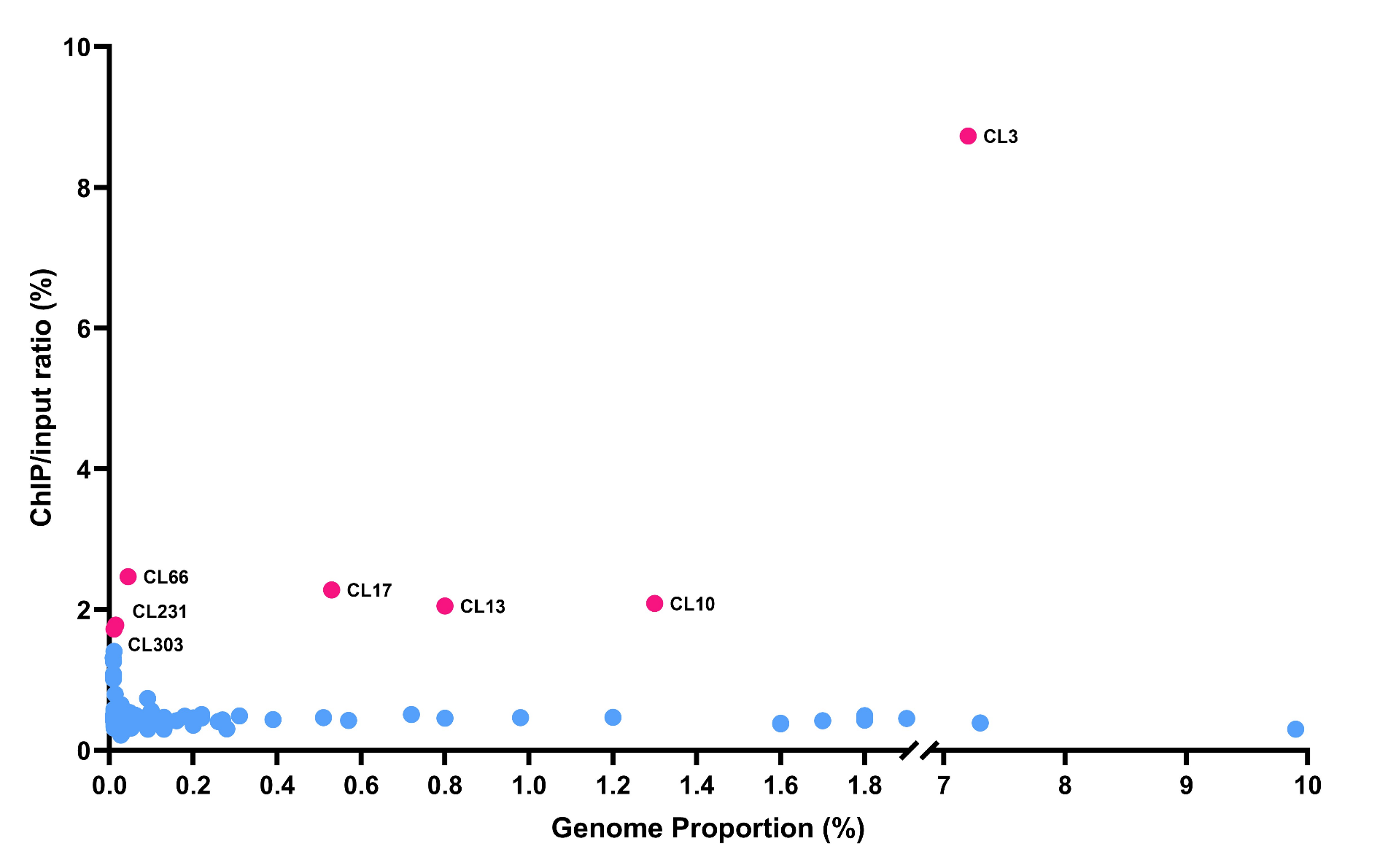


**Figure S3.** Enrichment and annotation of the repeat clusters in ChIP-seq data. Repeat clusters are represented by dots. The *x*-axis is the genome percentage for each cluster. The *y*-axis is the ratio of ChIP-seq to input reads, which represents the enrichment of the corresponding cluster in the ChIP-seq data.


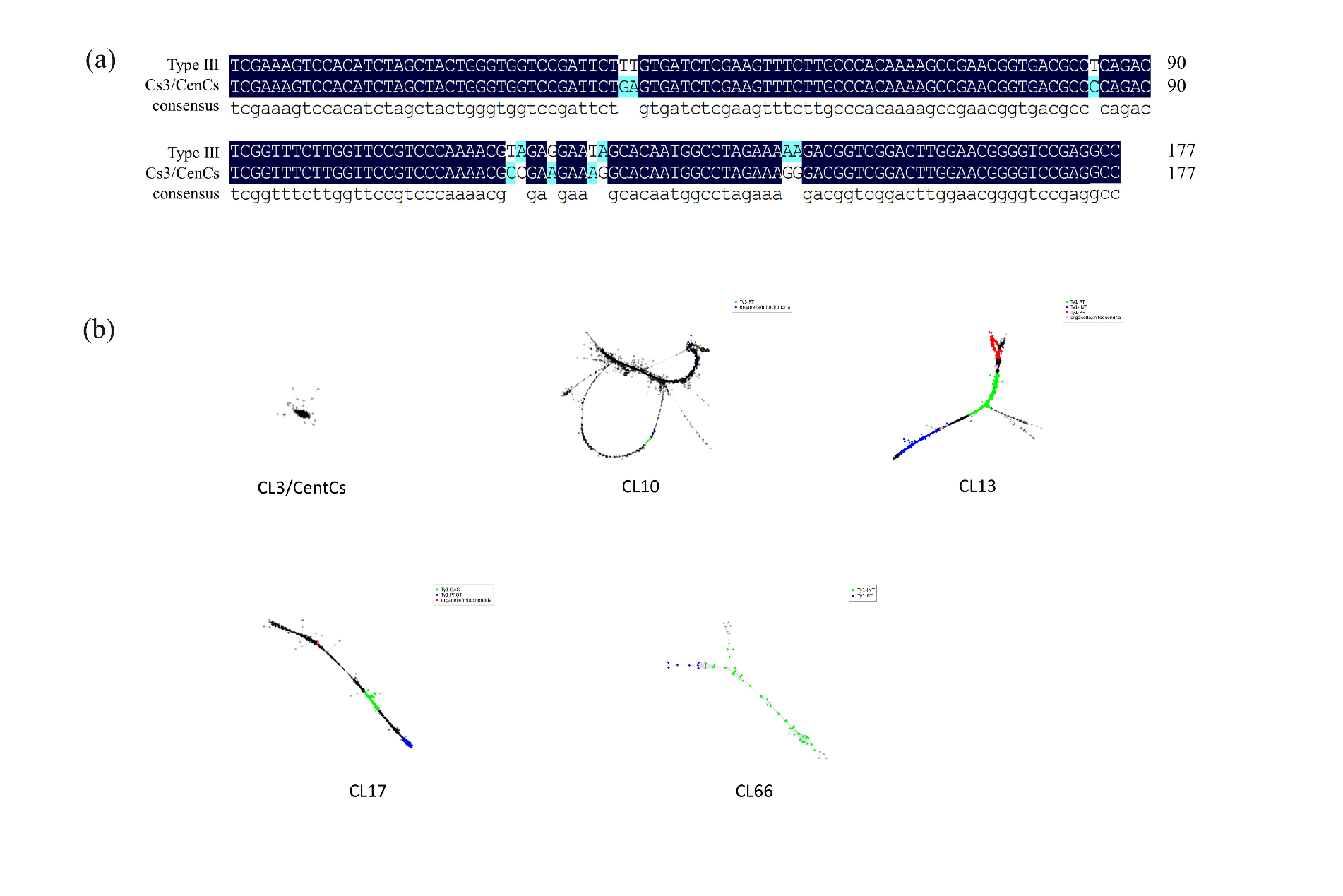


**Figure S4**. Alignment between Cs3 (CentCs) and type Ⅲ (a) and annotation of the repeat clusters in ChIP-seq data (b). Sequence similarities to those clusters conserved retrotransposon protein domains are highlighted by different colors (b).


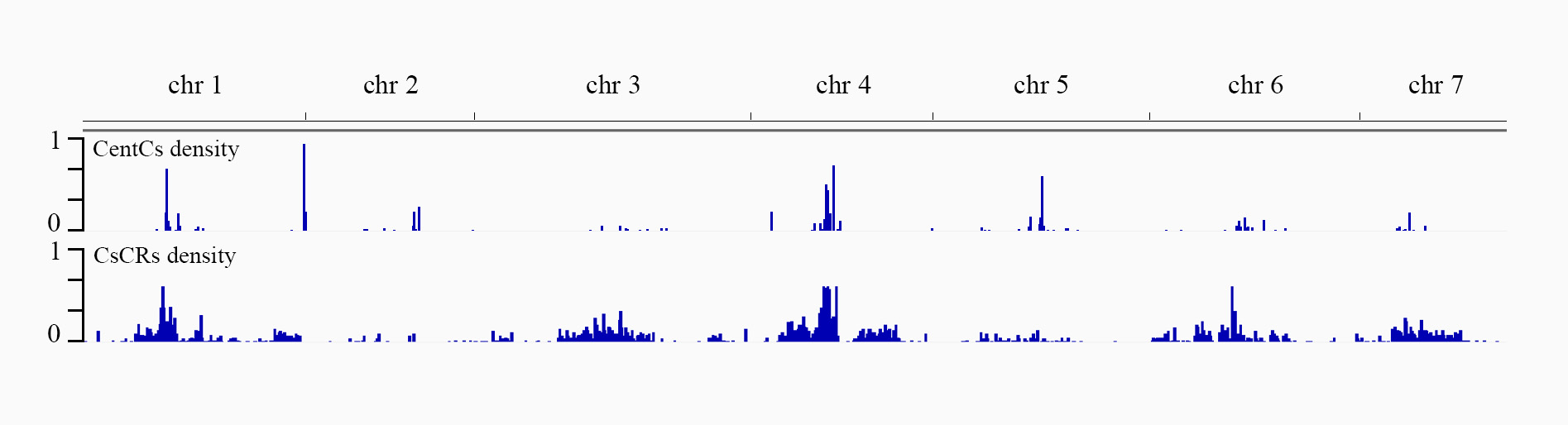


**Figure S5.** Chromosome distribution of CentCs and CsCRs.


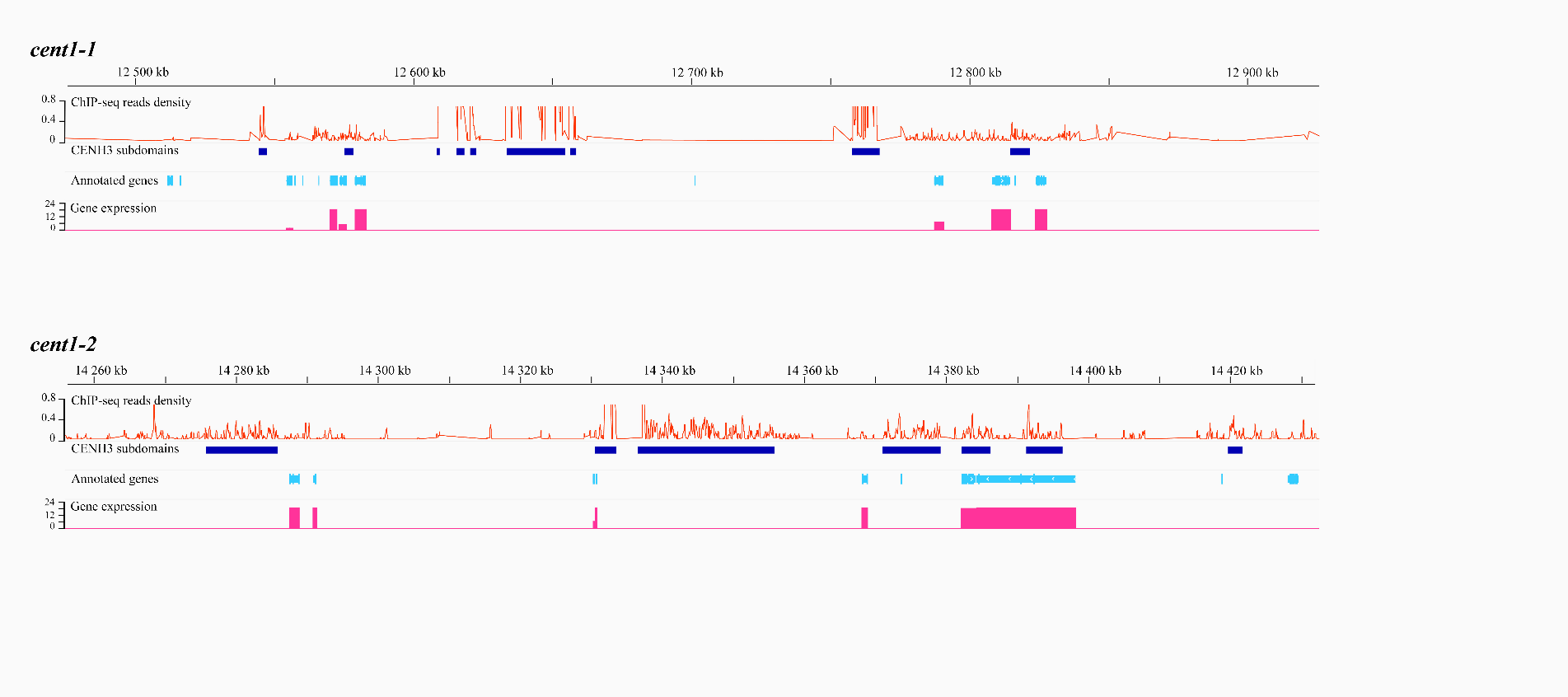


**Figure S6.** Localization of CsCENH3 binding domains and gene expression in the centromere 1.

The top track of each panel indicates the position on the corresponding chromosome. ChIP-seq read densities were calculated in 1-kb windows and represented by line plots. CsCENH3 subdomains are represented by blue bars under ChIP-seq mapping. Annotated genes are shown in coordination with the cucumber genome (light blue). The pink vertical bar represents the number of tissues in which the gene is expressed (FPKM>1).


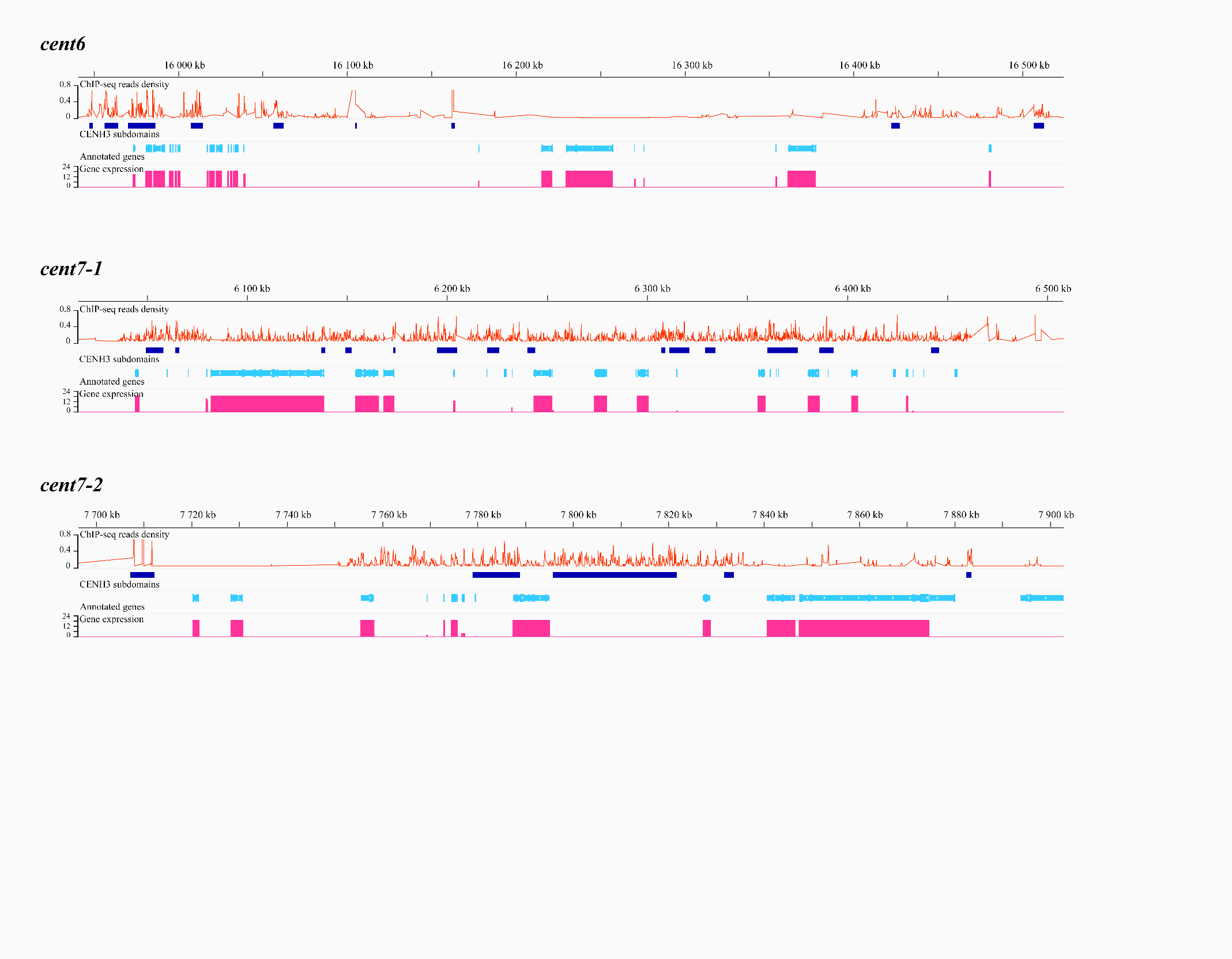


**Figure S7.** Localization of CsCENH3 binding domains and gene expression in the centromeres 6 and 7.

The top track of each panel indicates the position on the corresponding chromosome. ChIP-seq read densities were calculated in 1-kb windows and represented by line plots. CsCENH3 subdomains are represented by blue bars under ChIP-seq mapping. Annotated genes are shown in coordination with the cucumber genome (light blue). The pink vertical bar represents the number of tissues in which the gene is expressed (FPKM>1).


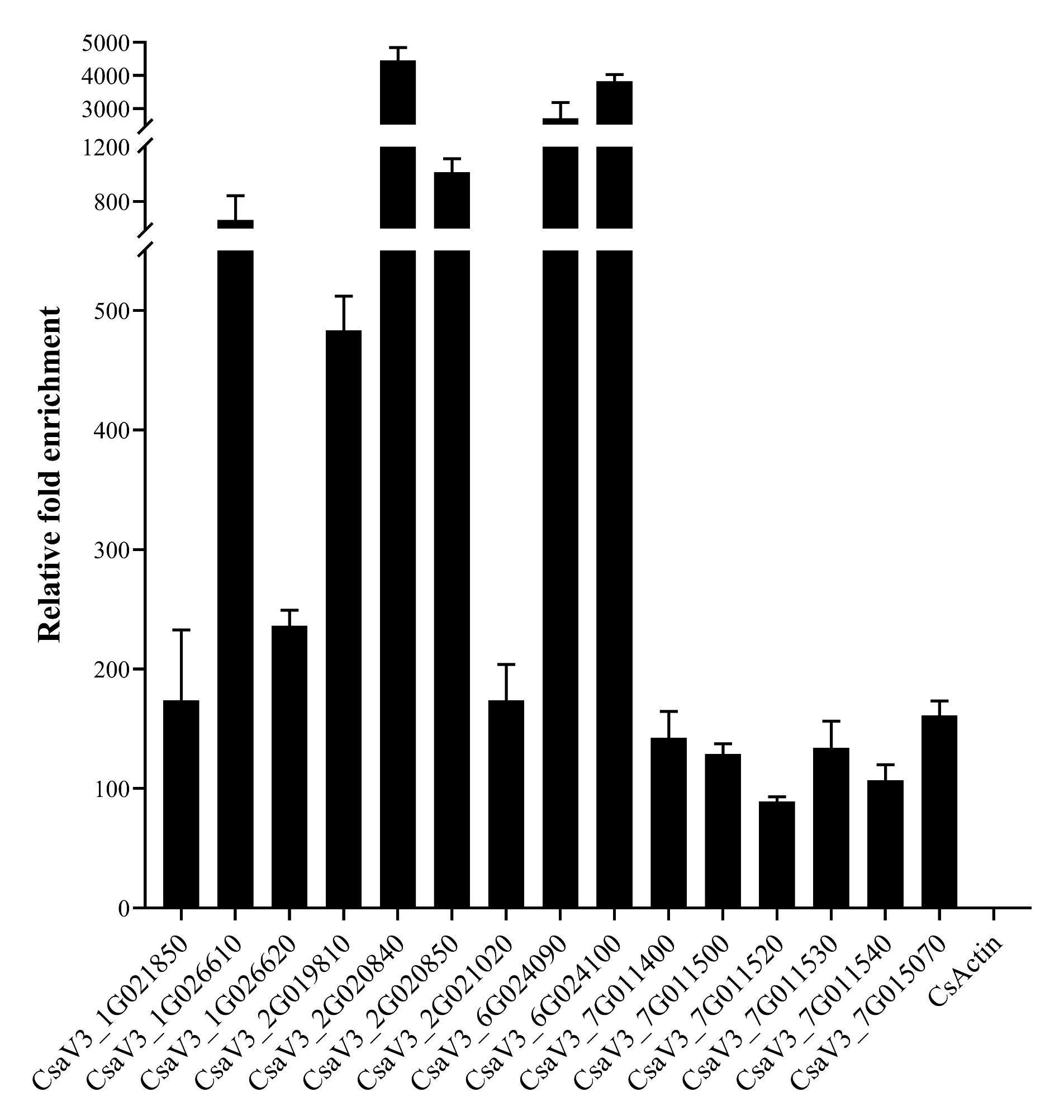


**Figure S8.** Relative fold enrichment (RFE) of genes in the CsCENH3 subdomain. ChIP-qPCR results show that 15 genes from ChIPed DNA (shown on the x-axis) were associated with CsCENH3. *CsActin* located in non-centromeric regions serves as a negative control and shows a small amount of enrichment. IgG and *CsActin* were used to normalize relative fold enrichment of genes. Error bars represent SD values from triplicates.


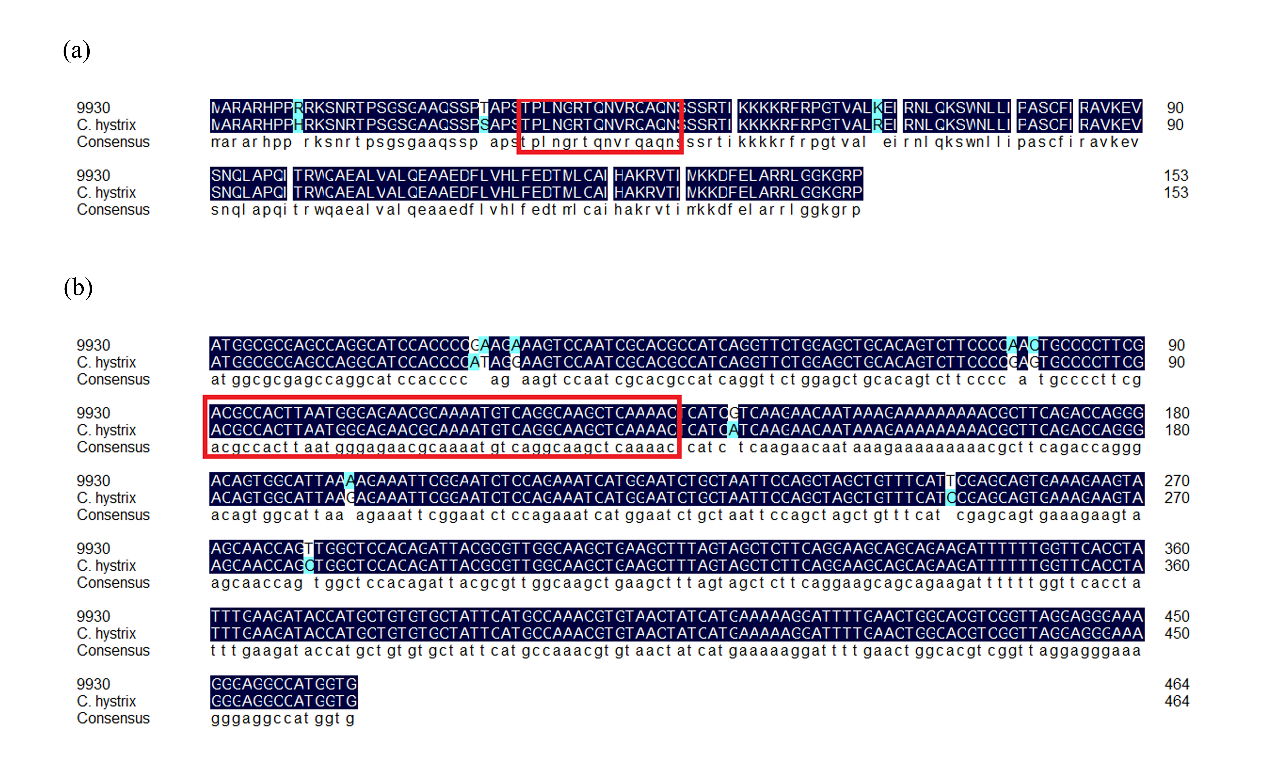


**Figure S9.** Amino acid sequence (a) and coding sequence (CDS) (b) alignments of CENH3 from cucumber cultivar ‘9930’ and *C. hystrix*. The peptide was used to generate anti-CsCENH3 antibodies are shown in red boxes.


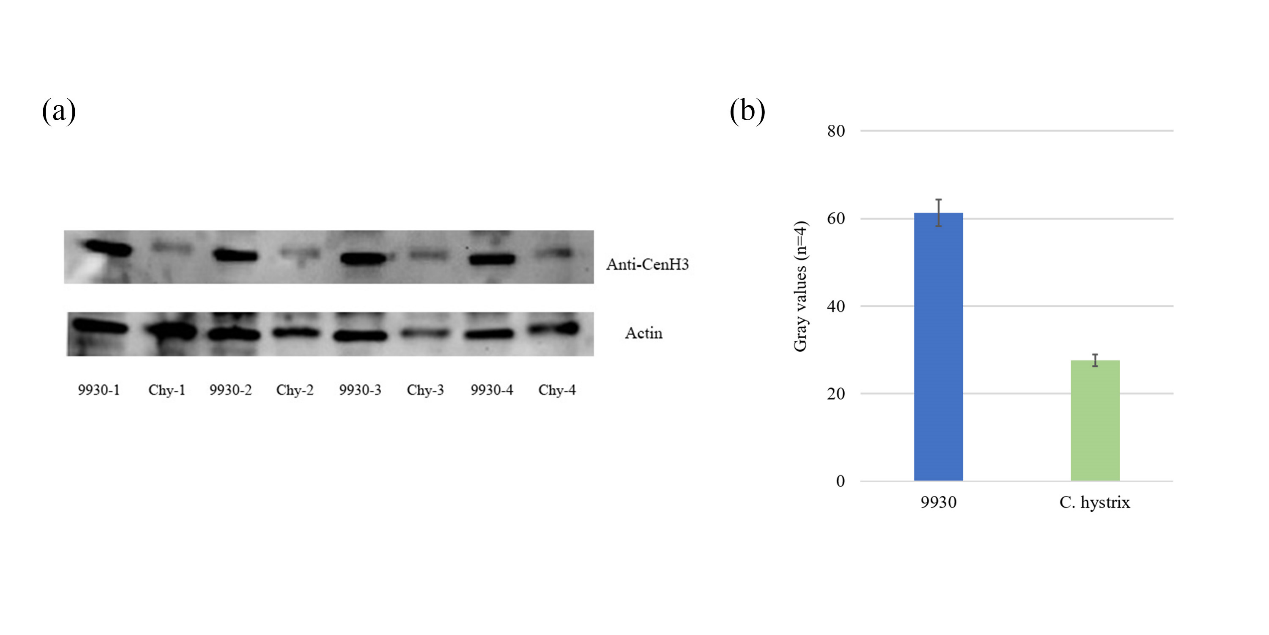


**Figure S10.** Western blotting assay (a) and gray value analysis (b) of CENH3 protein content between cucumber cultivar ‘9930’ and *C. hystrix*.
