# Supplementary figures and images for: Characterization of the CsCENH3 protein and centromeric DNA profiles reveal the structures of centromeres in cucumber
Source: Hortic Res. 2024 May 7;11(7):uhae127. doi: 10.1093/hr/uhae127 (PMC11220175; doi:10.1093/hr/uhae127)

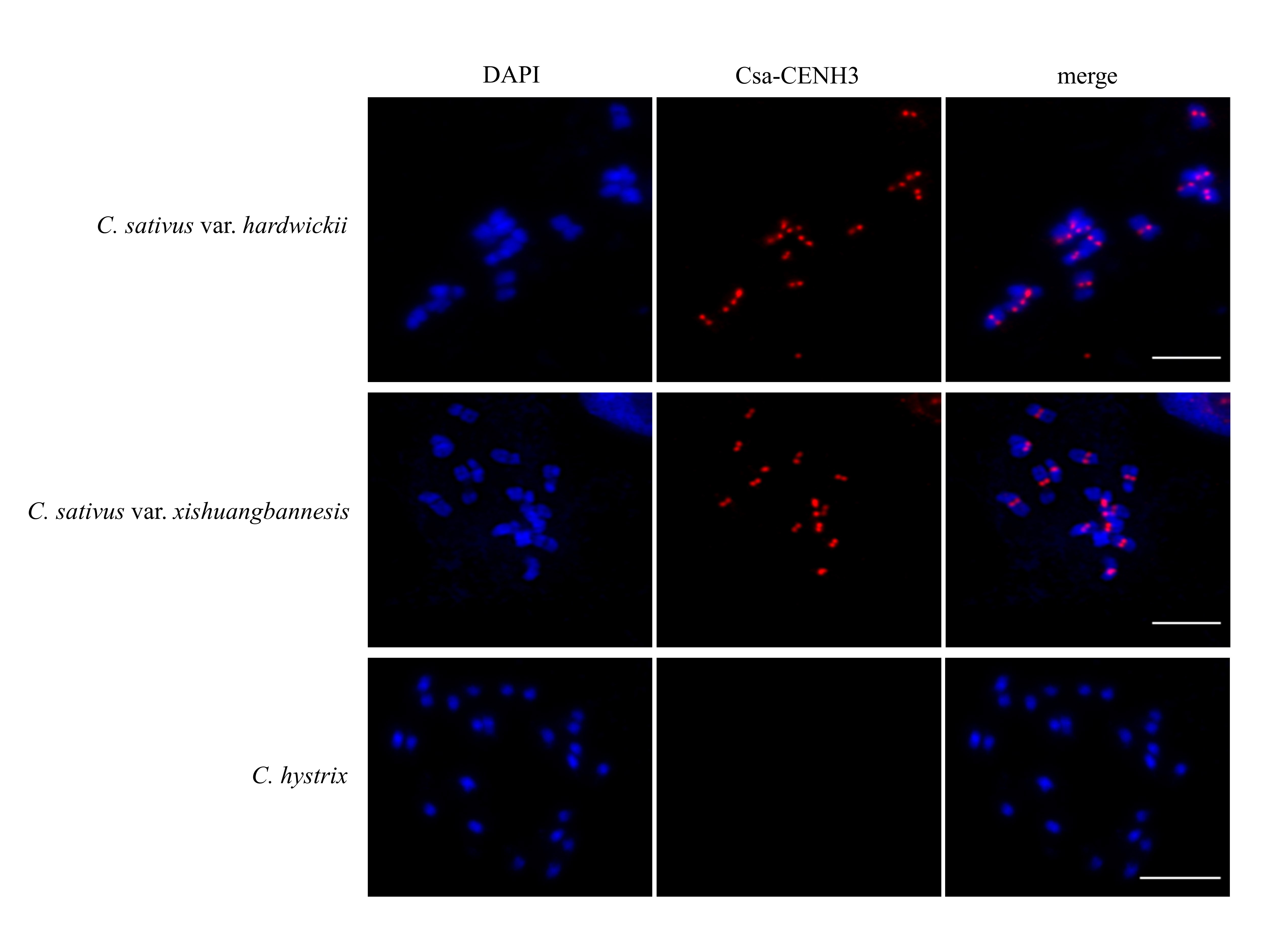

Supplement: Web_Material_uhae127 [file web_material_uhae127.zip › Fig. S1.tif]

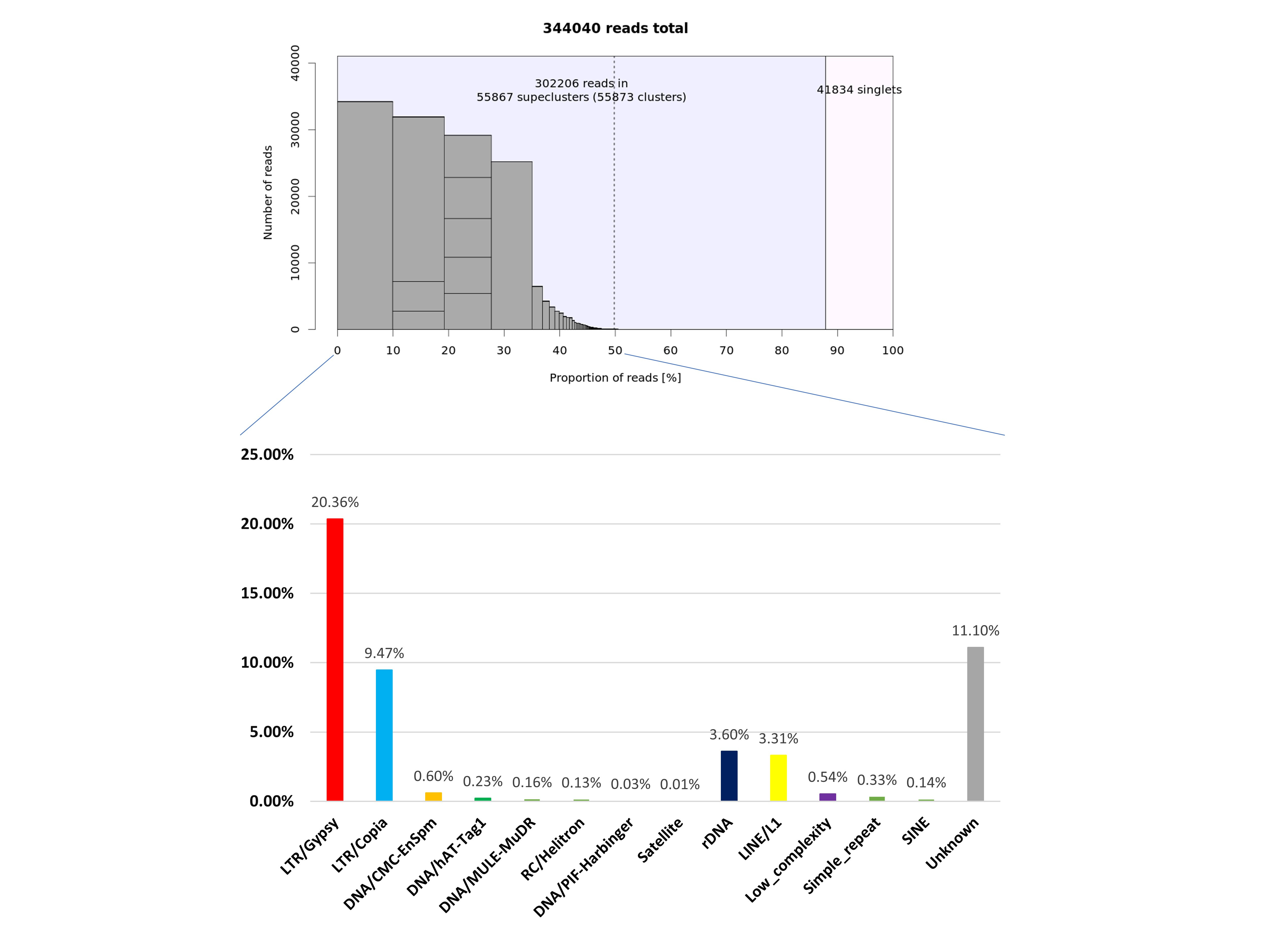

Supplement: Web_Material_uhae127 [file web_material_uhae127.zip › Fig. S2.tif]

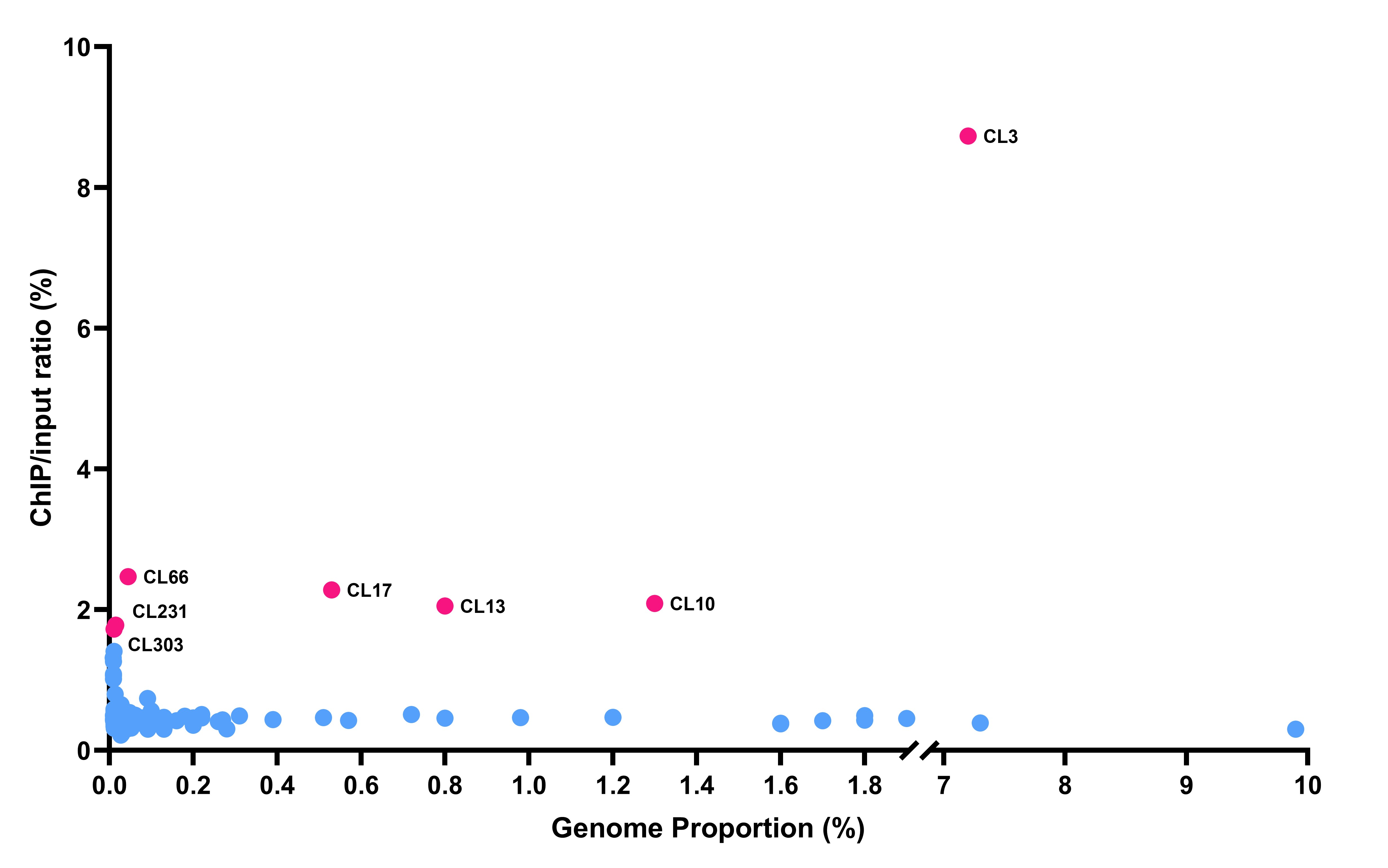

Supplement: Web_Material_uhae127 [file web_material_uhae127.zip › Fig. S3.tif]

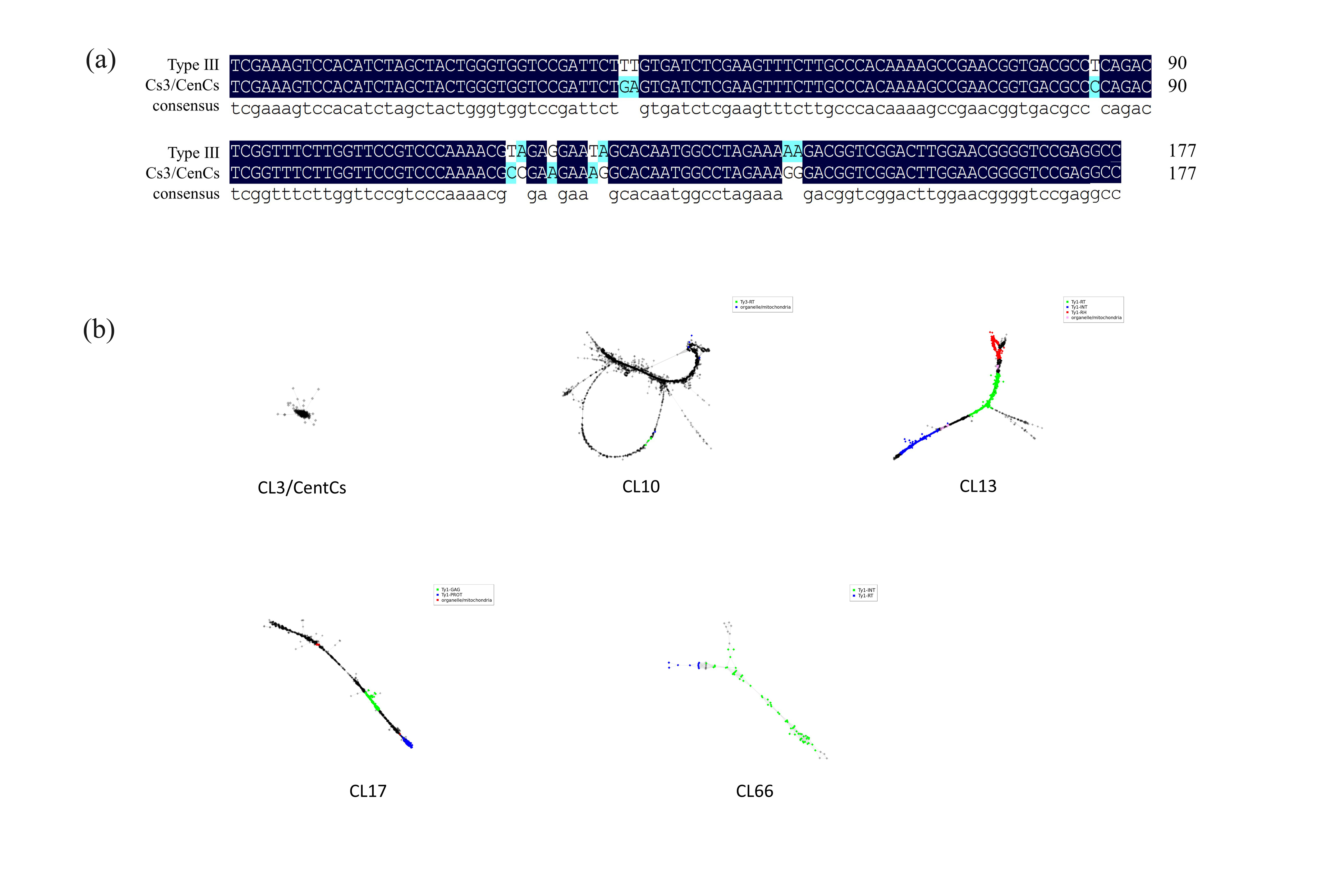

Supplement: Web_Material_uhae127 [file web_material_uhae127.zip › Fig. S4.tif]

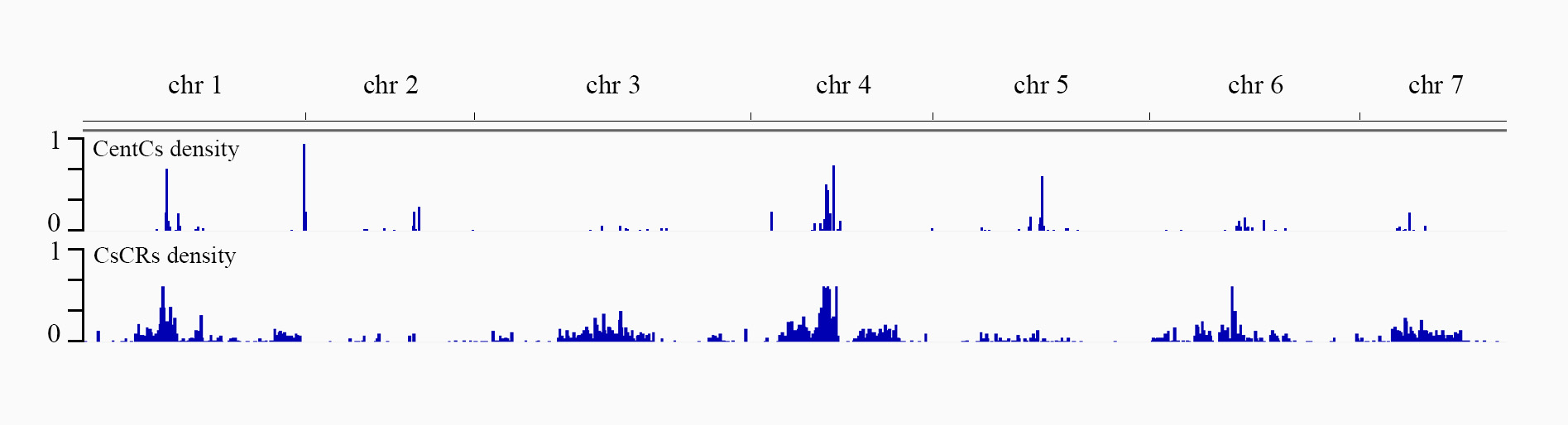

Supplement: Web_Material_uhae127 [file web_material_uhae127.zip › Fig. S5.tif]

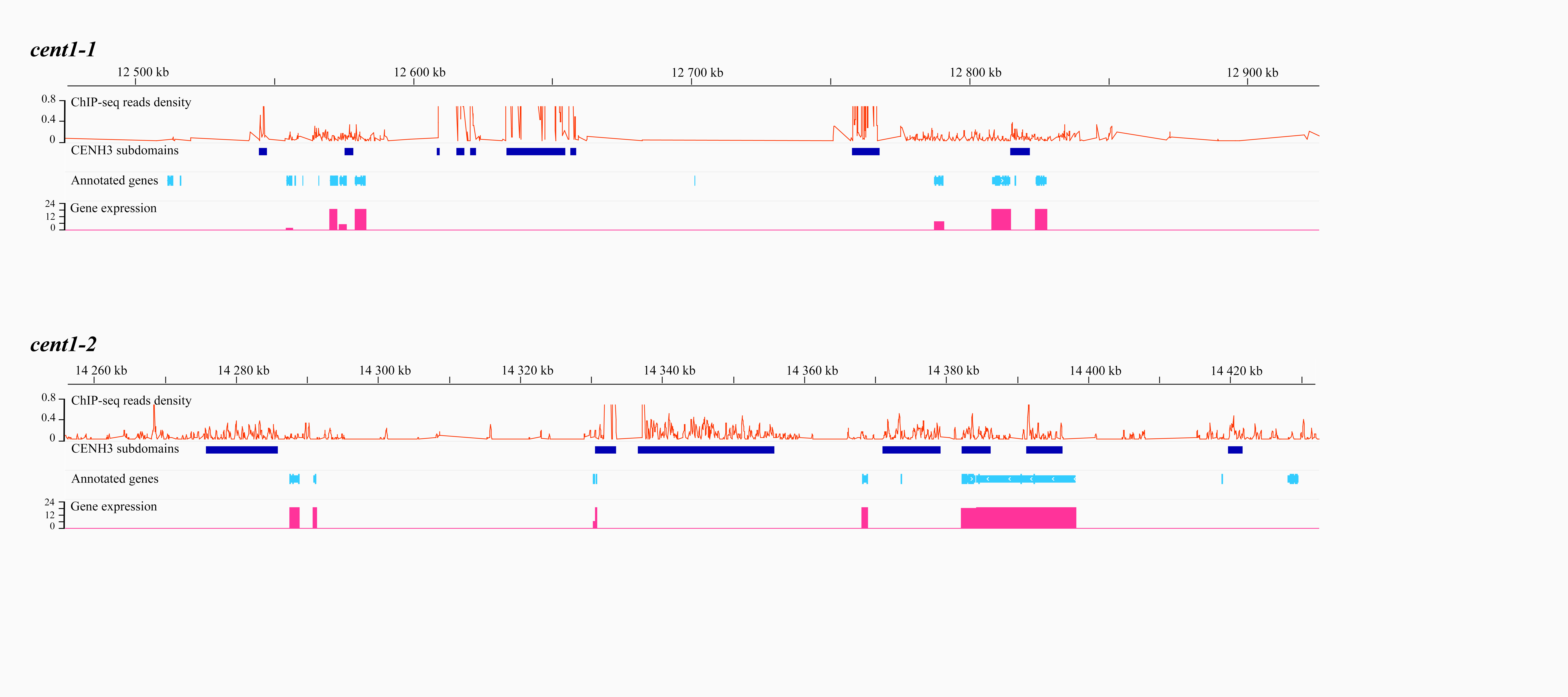

Supplement: Web_Material_uhae127 [file web_material_uhae127.zip › Fig. S6.tif]

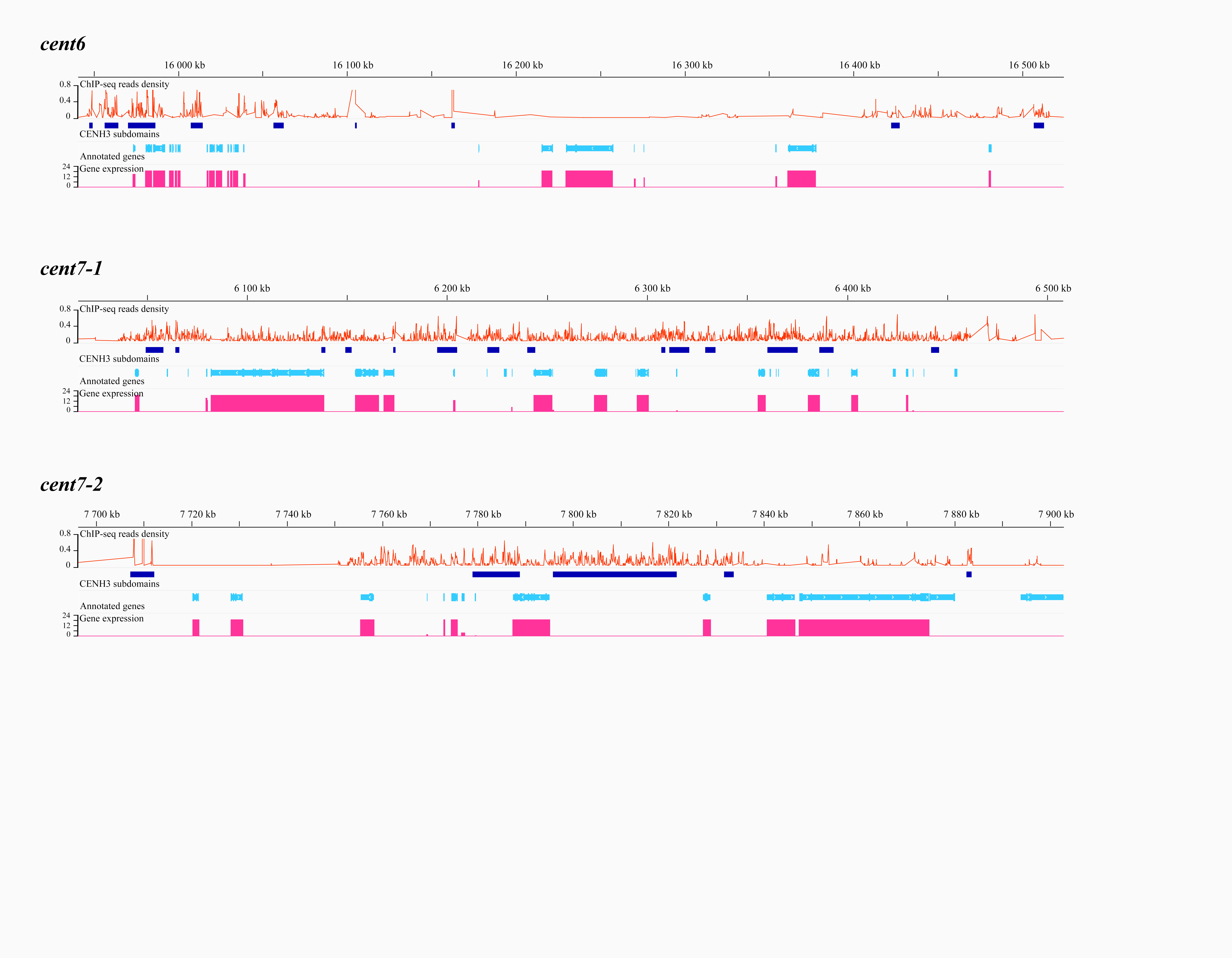

Supplement: Web_Material_uhae127 [file web_material_uhae127.zip › Fig. S7.tif]

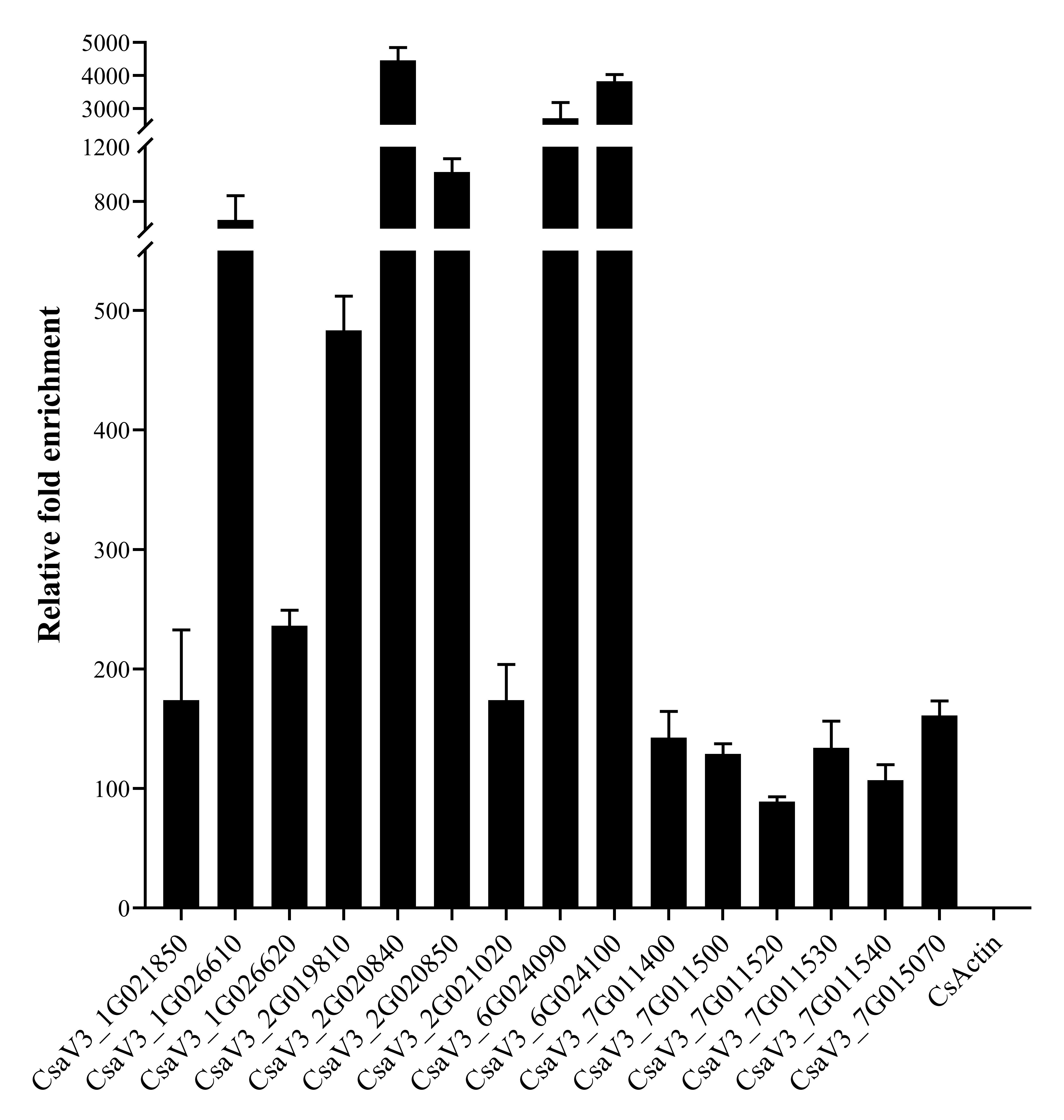

Supplement: Web_Material_uhae127 [file web_material_uhae127.zip › Fig. S8.tif]

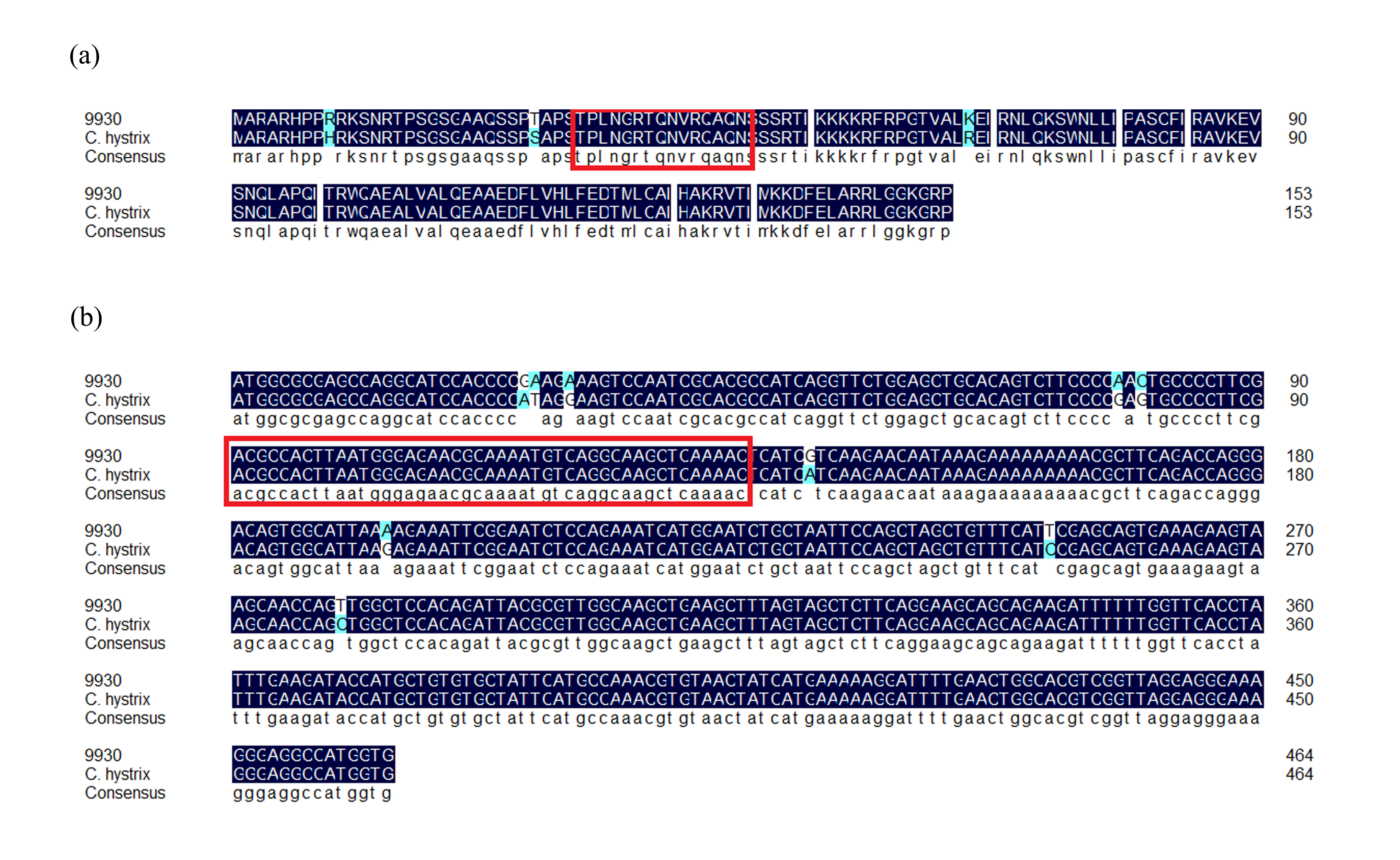

Supplement: Web_Material_uhae127 [file web_material_uhae127.zip › Fig. S9.tif]

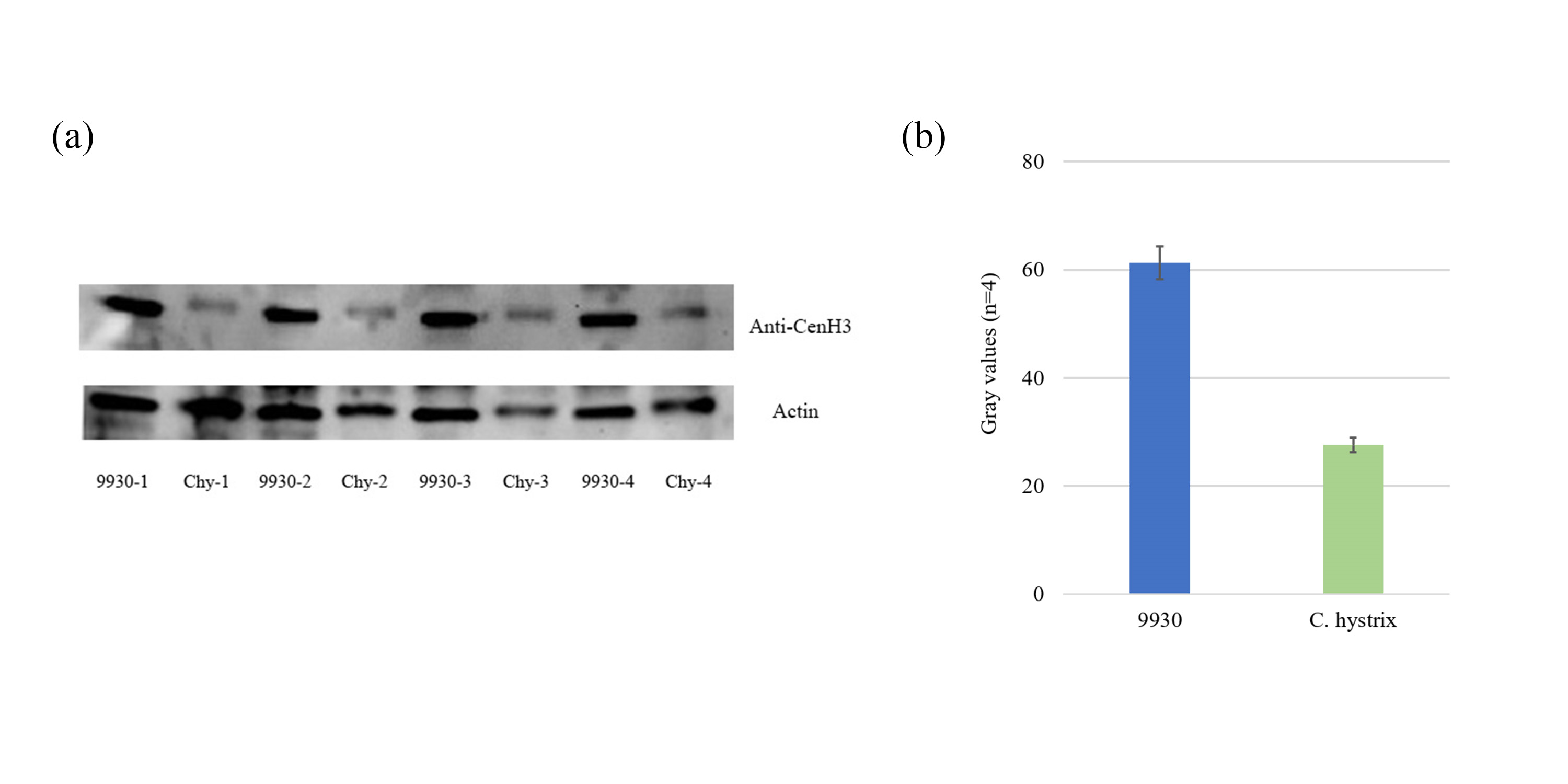

Supplement: Web_Material_uhae127 [file web_material_uhae127.zip › Fig. S10.tif]
